# Regulation of antigen-specific T cell infiltration and spatial architecture in multiple myeloma and premalignancy

**DOI:** 10.1172/JCI167629

**Published:** 2023-08-01

**Authors:** M. Hope Robinson, Nancy Y. Villa, David L. Jaye, Ajay K. Nooka, Alyssa Duffy, Samuel S. McCachren, Julia Manalo, Jeffrey M. Switchenko, Sierra Barnes, Sayalee Potdar, Maryam I. Azeem, Ava A. Horvat, Vaunita C. Parihar, Jingjing Gong, Yan Liang, Geoffrey H. Smith, Vikas A. Gupta, Lawrence H. Boise, Jonathan L. Kaufman, Craig C. Hofmeister, Nisha S. Joseph, Sagar Lonial, Kavita M. Dhodapkar, Madhav V. Dhodapkar

**Affiliations:** 1 Department of Hematology/Medical Oncology,; 2 Department of Pathology and Laboratory Medicine, and; 3 Winship Cancer Institute, Emory University, Atlanta, Georgia, USA.; 4 Aflac Cancer and Blood Disorders Center, Children’s Healthcare of Atlanta, Department of Pediatric Hematology/Oncology, Emory University, Atlanta, Georgia, USA.; 5 Pathology Department, NanoString Inc., Seattle, Washington, USA.

**Keywords:** Hematology, Immunology, Adaptive immunity, Cancer immunotherapy

## Abstract

Entry of antigen-specific T cells into human tumors is critical for immunotherapy, but the underlying mechanisms are poorly understood. Here, we combined high-dimensional spatial analyses with in vitro and in vivo modeling to study the mechanisms underlying immune infiltration in human multiple myeloma (MM) and its precursor monoclonal gammopathy of undetermined significance (MGUS). Clustered tumor growth was a feature of MM but not MGUS biopsies, and this growth pattern was reproduced in humanized mouse models. MM biopsies exhibited intralesional as well as spatial heterogeneity, with coexistence of T cell–rich and T cell–sparse regions and the presence of areas of T cell exclusion. In vitro studies demonstrated that T cell entry into MM clusters was regulated by agonistic signals and CD2-CD58 interactions. Upon adoptive transfer, antigen-specific T cells localized to the tumor site but required in situ DC–mediated antigen presentation for tumor entry. C-type lectin domain family 9 member A–positive (CLEC9A^+^) DCs appeared to mark portals of entry for gradients of T cell infiltration in MM biopsies, and their proximity to T cell factor 1–positive (TCF1^+^) T cells correlated with disease state and risk status. These data illustrate a role for tumor-associated DCs and in situ activation in promoting the infiltration of antigen-specific T cells in MM and provide insights into spatial alterations in tumor/immune cells with malignant evolution.

## Graphical abstract

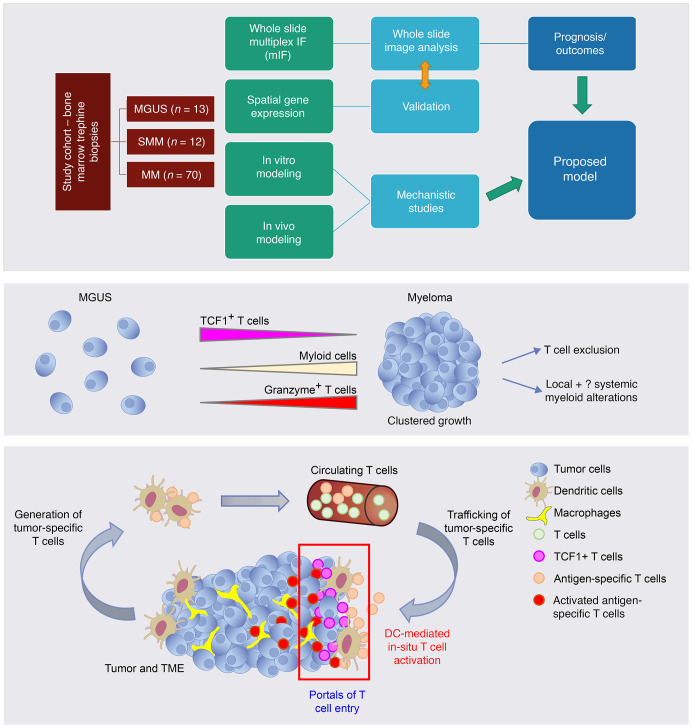

## Introduction

The degree and nature of T cell infiltration into tumors has emerged as an important determinant of response to immune therapy (1). Several studies have characterized spatial aspects of T cell infiltration in solid tumors and linked it to outcomes (1). In contrast to solid tumors, premalignant cells in hematologic malignancies are already widely disseminated. How the spatial aspects of tumor-immune architecture change with malignant evolution and the mechanisms underlying the entry of antigen-specific T cells in hematologic malignancies are poorly understood.

Multiple myeloma (MM) is a common hematologic malignancy characterized by the growth of malignant plasma cells (PCs) in the bone marrow. MM is universally preceded by precursor lesions termed monoclonal gammopathy of undetermined significance (MGUS) or smoldering multiple myeloma (SMM) (2). Prior studies have demonstrated the presence of tumor-specific T cells in the marrow of patients with MGUS or MM (3, 4). However, these and most other studies of the tumor microenvironment (TME) in MM have to date been largely based on analyses of bone marrow aspirates (as opposed to biopsies) and therefore did not consider spatial aspects of tumor growth or immune cell infiltration (5). The mechanisms underlying the entry of antigen-specific T cells into MM tumors are also largely unknown. T cell redirection therapies have led to impressive tumor regressions and begun to dramatically alter the therapeutic landscape in MM (6, 7). The clinical success of these therapies underscores the need to understand the spatial aspects of immune infiltration and the mechanisms regulating immune cell entry in MM lesions.

In this study, we combined high-dimensional spatial analyses with in vitro (8) and in vivo modeling of human MM (9) tumors and their precursor states to gain mechanistic insights into the regulation of immune infiltration and patterns of tumor growth (Supplemental Figure 1; supplemental material available online with this article; https://doi.org/10.1172/JCI167629DS1).

## Results

### Immune composition in MGUS, SMM, and MM.

In order to better understand the immune composition and spatial aspects in the context of the evolution of malignancy, we used multiplex immunofluorescence (mIF) to study bone marrow biopsies from 95 patients with PC malignancy or its precursor states (MGUS *n* = 13; SMM *n* = 12; MM *n* = 70) (see staining panels 1–3 in Supplemental Figure 2). Clinical characteristics of patients with MM are provided in Supplemental Table 1. As expected, SMM and MM bone marrows exhibited increased CD138^+^ PC density relative to MGUS (Supplemental Figure 3A). The mean density of CD3^+^ T cells was similar between the cohorts (Supplemental Figure 3B), whereas the mean expression of CD68, a myeloid marker, progressively increased from MGUS to MM (Supplemental Figure 3C). We have previously shown that bone marrow aspirates from patients with MGUS contain an increased proportion of TCF1^+^ stem-like memory T cells (10). MM development is characterized by progressive attrition of these cells and, instead, a higher proportion of granzyme B^+^ (GZMB^+^) terminal effector T cells in bone marrow aspirates (10). Consistent with these studies, the density of TCF1^+^ T cells was increased in MGUS and SMM biopsies relative to MM biopsies (Supplemental Figure 4A). The density of total CD8^+^ T cells as well as of CD8^+^GZMB^+^ T cells was also higher in MM biopsies relative to MGUS and SMM biopsies (Supplemental Figure 4, B and C). In contrast, the density of CD4^+^ T cells and CD4^–^CD8^–^GZMB^+^ cells (consistent with NK cells) was comparable between cohorts (Supplemental Figure 4, D and E). These cohorts also did not differ in terms of density of CD4^+^FOXP3^+^ Tregs (Supplemental Figure 4F). Together, these data demonstrate that, while there was some variance in the immune composition within each of the cohorts, there were distinct differences in the immune composition of marrow trephine biopsies, with an increase in effector cells and a decline in stem-like T cells in MM biopsies versus MGUS biopsies.

### Spatial aspects of immune infiltration.

**Figure 1:**
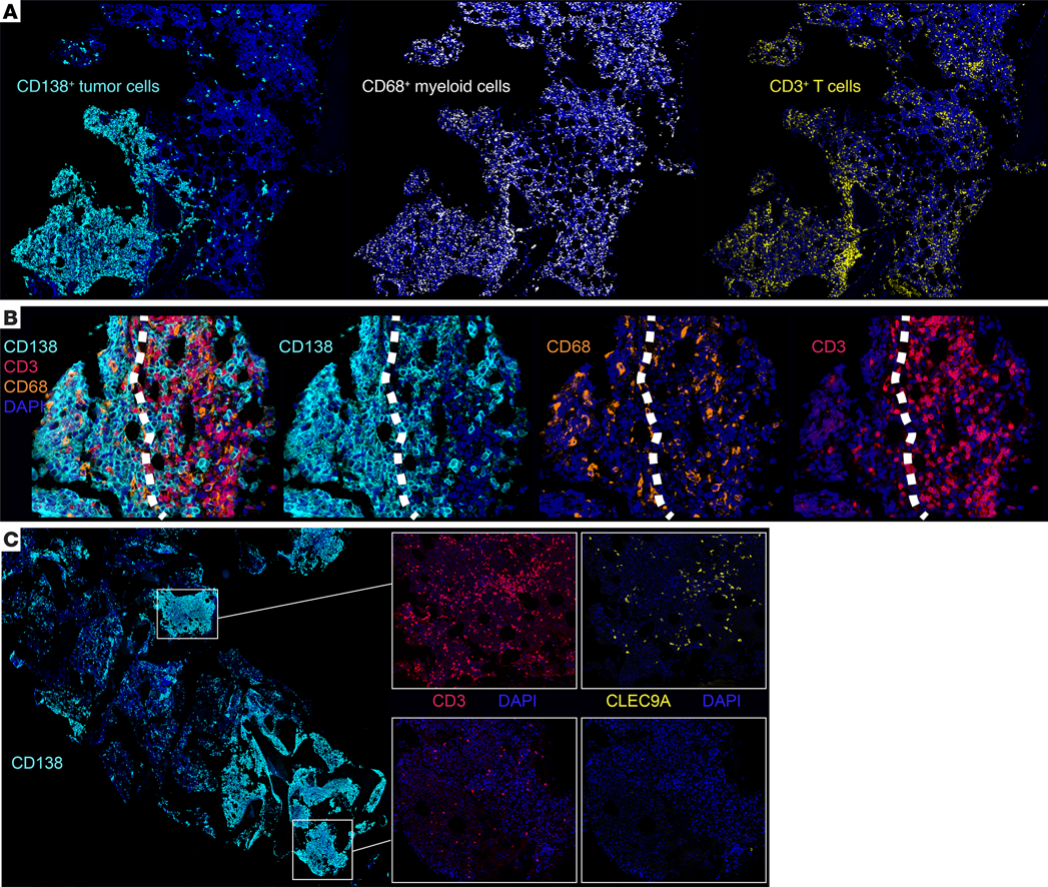
Spatial heterogeneity of immune infiltration in MM. mIF staining was performed on FFPE sections from 95 patient samples (MGUS *n* = 13; SMM *n* = 12; MM *n* = 70) (see Methods and Supplemental Figure 2 for staining panels). (**A**) Representative IHC images (original magnification, ×4) showing the patchy nature of the CD138^+^ tumor infiltration compared with the diffuse infiltration of CD68^+^ myeloid cells and the nonuniform pattern of T cell infiltration. (**B**) T cell exclusion: Representative IHC images (original magnification, ×30) showing accumulation of T cells at the tumor edge, but infiltration of CD68^+^ myeloid cells. White dotted lines indicate the tumor edge. (**C**) Intralesional heterogeneity showing T cell–rich and T cell–poor areas coexisting in the same biopsy specimen. T cell–rich hotspots are associated with infiltration of CLEC9A^+^ DCs. Original magnification, ×2 (lower-powered view) and ×14 (insets).

Prior studies have shown that a distinct feature of MM biopsies is the patchy accumulation of tumors, with the formation of focal micro-clusters (11), which we also observed in the current study (Supplemental Figure 5A). We did not observe such micro-cluster formation in the MGUS biopsies. In order to quantify MM cluster formation, we utilized image analysis and machine learning to quantify the maximum number of CD138^+^ cells within a 1,000 mm radius of each tumor cell, as an indirect measure of tumor clustering. This CD138^+^ proximity analysis demonstrated that MM was characterized by higher numbers of proximate CD138^+^ cells within a 1,000 μM radius, relative to MGUS and SMM, even when MM specimens with a high PC burden (PC infiltration >30%) were excluded (Supplemental Figure 5B). Similar results were obtained when a smaller radius (100 mm) was considered (Supplemental Figure 5C). Multiplex imaging was performed to evaluate the impact of clustered tumor growth on immune infiltration. In contrast to patchy infiltration by tumor cells, CD68^+^ myeloid cells were diffusely distributed throughout the marrow in MM, including in areas with low tumor infiltration, whereas the pattern of T cell infiltration was relatively nonuniform (Figure 1A and H&E images in Supplemental Figure 6). This was also reflected in a higher standard deviation for intercellular distances between T cells compared with that between CD68^+^ myeloid cells (Supplemental Figure 7). In some cases, T cells were found to accumulate at the edge of tumor clusters, reminiscent of the pattern of T cell exclusion described in solid tumors (Figure 1B and Supplemental Figure 8) (12). However, we found that CD68^+^ myeloid cells readily infiltrated these clusters. Importantly, the patterns of T cell infiltration could be heterogeneous within the same biopsy, with T cell rich areas coexisting with other areas showing a paucity of T cells (Figure 1C). The T cell–rich areas were almost always associated with CLEC9A^+^ DCs, while T cell–poor regions lacked these DCs (Figure 1C and Supplemental Figure 9). The finding of regions of T cell exclusion was a feature of MM but not MGUS, as the latter lacked tumor micro-clusters. To test whether the observed tendency of human MM cells to grow in a multifocal fashion could be reproduced in mouse models, we evaluated the early growth of these cells in vivo as xenografts in humanized mice (9). Tumor cells from both MGUS and MM grow progressively in these mice (9). However, early growth of tumor cells recapitulated the pattern seen in patients, with early formation of clusters with MM but not MGUS cells at a stage when the amount of clonal Igs was comparable (Supplemental Figure 10). Together, these data illustrate extensive spatial heterogeneity in MM, with clustered tumor growth and distinct patterns of immune infiltration in MM.

### Mechanisms regulating T cell entry into MM tumors: role of agonistic signaling and CD2-CD58 interactions.

**Figure 2:**
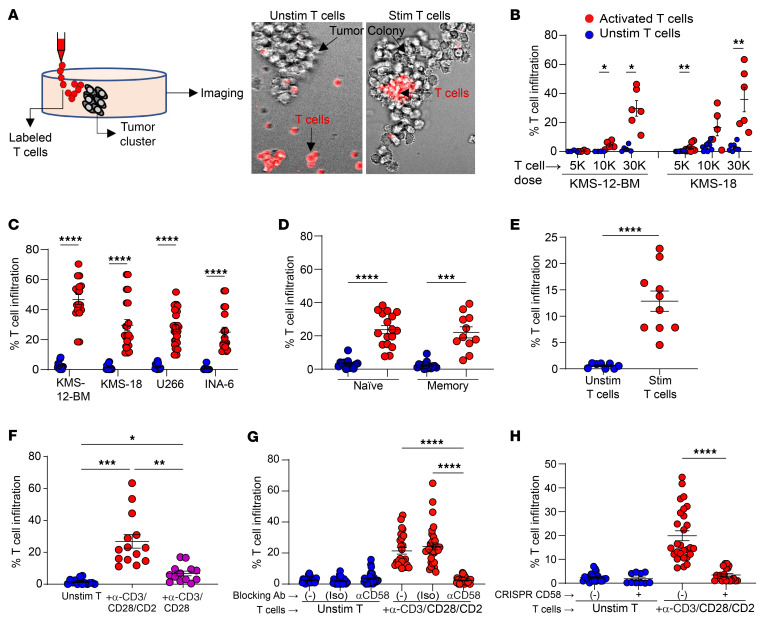
Entry of T cells into MM tumor clusters: effect of agonistic signaling and the CD2/CD58 axis. (A) Outline of the experimental model. Unstimulated (Unstim) and stimulated (Stim) T cells were placed adjacent to MM tumor clusters in methylcellulose. T cell infiltration was quantified as the proportion of the colony area infiltrated by fluorescence-labeled T cells. (B and C) Effect of preactivation of T cells with α-CD3/CD28/CD2 on T cell infiltration into tumor clusters. *n* = 4–28 clusters per condition. (B) Dose-dependent entry of T cells. (C) Entry into different MM cell line clusters. (D) Infiltration of naive versus memory T cells. Unstimulated or α-CD3/CD28/CD2–stimulated naive or memory T cells (*n* = 30,000 cells) were placed adjacent to KMS-18 clusters. *n* = 11–17. (E) Entry of autologous T cells into primary MM tumor clusters. Unstimulated or α-CD3/CD28/CD2–stimulated CD3^+^ bone marrow T cells (*n* = 100,000 cells) were placed adjacent to primary CD138^+^ clusters. *n* = 8–10. (F) Effect of preactivation with α-CD3/CD28 antibodies with or without α-CD2 antibody. *n* = 12–21. (G) Effect of α-CD58–blocking antibodies. U266 MM colonies were pretreated with α-CD58 or IgG1κ isotype control (Iso) or were untreated (–) prior to addition of unstimulated or α-CD3/CD28/CD2–stimulated T cells. T cell infiltration was analyzed as in A. *n* = 22–34. (H) Effect of CRISPR-mediated knockdown of CD58. Panel shows infiltration of unstimulated or α-CD3/CD28/CD2–stimulated T cells in U266 MM cells with or without CRISPR-mediated CD58 knockdown. *n* = 10–28. Data in B–H show the mean ± SEM. Each dot represents a distinct tumor cluster, and data were pooled from a minimum of 3 replicate experiments. *P < 0.05, **P < 0.01, ***P < 0.001, and ****P < 0.0001, by Brown-Forsythe and Welch’s ANOVA test with Dunnett’s T3 multiple-comparison test (D and F–H) and Mann-Whitney *U* test (B, C, and E).

Interactions between immune cells and MM or MGUS have to date been mostly studied in suspension cultures or analyses of bone marrow aspirates (2). As the growth of tumors in the form of focal lesions or micro-clusters emerged as a distinct feature of MM, we developed an in vitro model to test the entry of T cells into MM tumor clusters. For these studies, we first cultured MM cells in methylcellulose to generate tumor clusters and then tested the capacity of T cells to enter these colonies, when mechanically injected adjacent to these colonies (Figure 2A). Tumor colonies from MM cell lines were relatively resistant to the entry of unstimulated allogeneic T cells. However, ex vivo activation of T cells with anti-CD3/anti-CD28/anti-CD2 (α-CD3/CD28/CD2) antibodies led to dose-dependent entry of T cells into MM clusters (Figure 2, B and C). Ex vivo T cell activation promoted comparable entry of both naive and memory T cells (Figure 2D) into KMS-18 MM clusters. Ex vivo activation was also required for entry of bone marrow–derived T cells from patients with MM into clusters of autologous primary MM cells (Figure 2E). Analysis of single agonistic antibodies (and combinations) revealed that T cell entry of KMS-18 clusters was dependent on the degree and nature of stimulation and was higher for α-CD3/CD28/CD2–mediated stimulation relative to α-CD3/CD28 (Figure 2F), suggesting a potential role for CD2 in regulating T cell entry into MM clusters. CD2 on T cells may interact with CD58, which is known to be commonly expressed on MM cells (13). To further evaluate the role of CD2-CD58 interactions in T cell entry, we pretreated U266 MM cells with CD58-blocking antibodies. Antibody-mediated blockade of CD2-CD58 interactions abrogated the entry of activated T cells into MM clusters (Figure 2G). CRISPR-mediated knockdown of CD58 in MM cells also led to a reduction of T cell infiltration (Figure 2H). Together, these data demonstrate that the entry of T cells into MM tumor clusters was regulated by the nature and degree of agonistic signaling and depended on CD2-CD58 interactions.

### Mechanisms regulating the entry of antigen-specific T cells into MM tumors: role of DC-mediated in situ antigen presentation.

**Figure 3:**
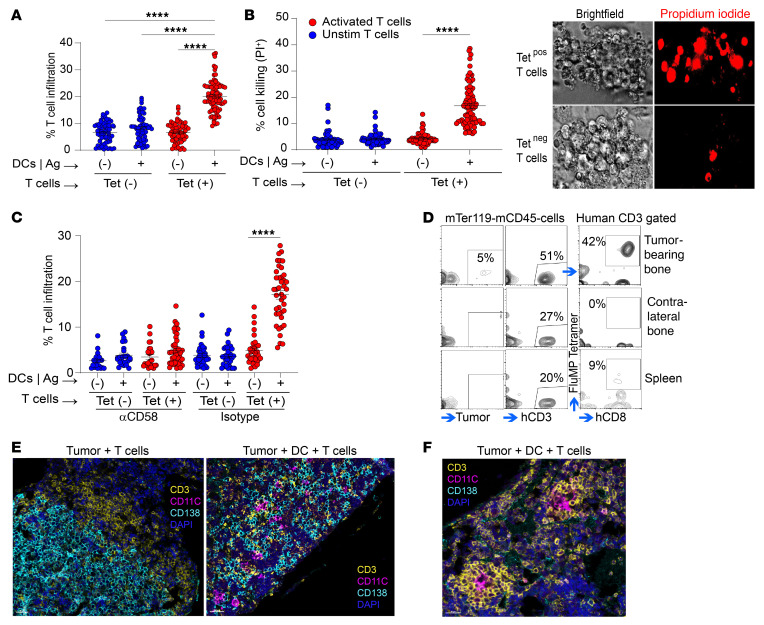
DC-mediated antigen presentation and T cell entry. (**A**–**C**) Effects of DCs on in vitro T cell infiltration. U266-MP colonies were injected with MP-pulsed HLA-A2^+^ mo-DCs (or unpulsed DCs as controls) followed by injection of MP-specific HLA-A2-tetramer^+^ T cells (tetramer^–^ T cells as a control) after 4 hours. T cell entry was quantified after overnight culture. For some experiments clusters were also labeled with PI to assess tumor cell lysis. (**A**) Effect of DC-mediated antigen (Ag) presentation on entry of antigen-specific T cells. *n* = 59–85. (**B**) PI staining showing killing of MM colonies. *n* = 54–89. (**C**) Effect of CD58 blockade on entry of antigen-specific T cells. *n* = 21–44. Data show the mean ± SEM. Each dot represents a distinct tumor cluster, and data were pooled from a minimum of 3 independent experiments. ****P < 0.0001, by Kruskal-Wallis test with Dunn’s multiple-comparison test (**A**–**C**). (**D**–**F**) Effects of DCs on in vivo T cell infiltration. U266-MP-rluc cells were first engrafted intrafemorally into MISTRG6 mice either as tumor cells alone, or with MP-pulsed, mo-DCs from an HLA-A2^+^ donor. Tumor growth was documented by IVIS, and mice were injected with T cells from the same donor, which had been previously expanded ex vivo using MP-pulsed DCs. mIF images are representative of 5 experiments with 3–7 mice per group. (**D**) Flow cytometric analysis showing selective enrichment of A2-MP-tetramer^+^ T cells at the tumor site. Note that most of the T cells in the spleen are tetramer^–^. (**E**) IHC images showing that T cells localizing to the tumor site did not efficiently enter the tumor clusters in tumors without human DCs (original magnification, ×24), but these T cells did so when the tumors contained DCs (original magnification, ×20). (**F**) Representative T cell clusters in bones of mice with tumor-associated DCs (original magnification, ×39.3). Tet, tetramer.

Neoantigen targeting strategies are being actively investigated across several human tumor types (14). In order to evaluate the entry of human antigen–specific T cells into tumors, we used HLA-A2–restricted immunodominant influenza matrix peptide (MP) as a model antigen (15). For these studies, HLA-A2^+^ U266 MM cells were engineered to express A2-restricted MP epitope (GILGFVFTL). MP-expressing U266 cells (U266-MP) readily presented antigen to MP-specific T cells in suspension cultures (Supplemental Figure 11). Next, U266-MP cells were grown in methylcellulose, and antigen-bearing U266-MP clusters were tested for entry of sorted MP-restricted HLA-A2-tetramer^+^ T cells. Surprisingly, we found that the addition of tetramer^+^ T cells alone to clusters of antigen-expressing MM cells led to minimal infiltration of antigen-specific T cells into antigen-expressing tumor clusters (Figure 3A). However, when MP-pulsed DCs were added to these clusters to provide in situ antigen–specific activation by professional antigen-presenting cells (APCs), it led to clear infiltration of antigen-specific (tetramer^+^) T cells, but not tetramer^–^ T cells, into tumor clusters (Figure 3A). This entry of antigen-specific T cells was associated with enhanced killing of target tumor cells (Figure 3B). To further evaluate whether the CD2/CD58 axis discussed earlier is also important for entry of antigen-specific T cells, we repeated these experiments in the presence of α-CD58–blocking antibody. Antibody-mediated blockade of CD58 led to inhibition of entry of antigen-specific T cells (Figure 3C). Similar findings were observed when we used XG-1, another HLA-A2^+^ MM cell line, which was also engineered to express MP (Supplemental Figures 12 and 13). Expression of CD58 on these MM cell lines was confirmed by flow cytometry (Supplemental Figure 14). Blockade of antigen presentation by DCs using treatment with α-MHCI antibody inhibited the entry of T cells (Supplemental Figure 15). Together, these data support a role for in situ DC–mediated antigen presentation in promoting entry of antigen-specific T cells into MM tumor clusters.

Upon adoptive transfer into mice bearing U266-MP-rluc tumor cells without DCs, MP-specific T cells localized to the tumor site, where they constituted the majority of the observed T cells (Figure 3D). In contrast, most of the T cells in the spleen were tetramer^–^. mIF analyses revealed that T cells at the tumor site in the bone marrow mostly accumulated at the edge of these tumors (Figure 3E). In contrast, when U266-MP tumors were coinjected with antigen-presenting DCs, MP-specific T cells could readily infiltrate tumor clusters (Figure 3E). Along with tumor clearing, mIF analysis of bones with human DCs also revealed areas of T cell clusters surrounding DCs (Figure 3F), reminiscent of the tertiary lymphoid structures seen in solid tumors. Together, these data suggest that DCs at tumor sites presenting tumor-specific antigens promote in situ activation of antigen-specific T cells and their entry into tumors.

**Figure 4:**
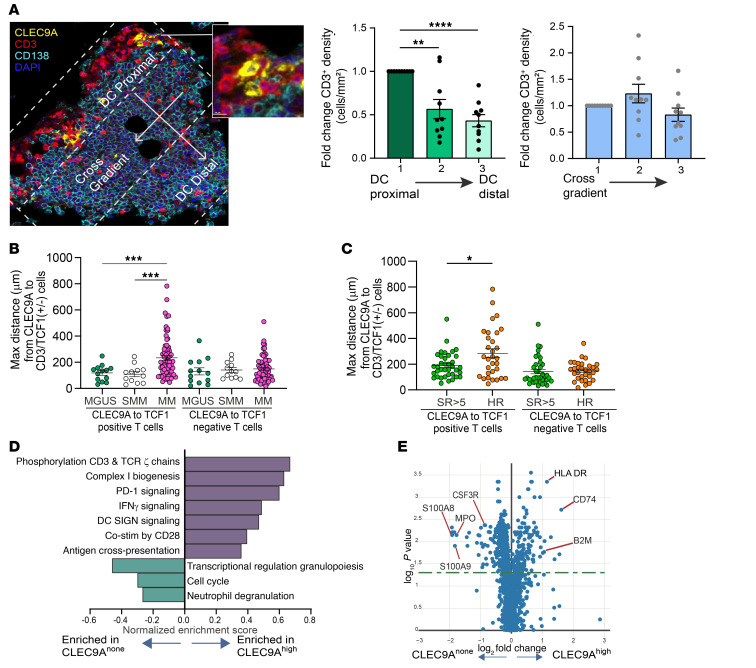
Spatial relationships between CLEC9A DCs and T cells. (**A**) CLEC9A^+^ DCs and T cell gradients. Representative IHC panel shows hotspot containing T cells and CLEC9A^+^ DCs (original magnification, ×20; inset, ×119). In order to quantify the relationship between DCs and T cell gradients within MM tumors, T cell density was measured in tumor clusters in regions proximal or distal to CLEC9A^+^ DCs. T cell density within a cross-gradient in the same cluster served as a control. Bar graphs represent the fold change (mean ± SEM) relative to the proximal zone. Significance was determined by repeated-measures 1-way ANOVA with the Geisser-Greenhouse correction and Dunnett’s multiple-comparison test, with individual variances computed for each comparison. Each dot represents a gradient zone in a ROI (*n* = 10 regions from 6 patient samples). (**B**) Proximity of CLEC9A^+^ DCs and TCF1^+/–^ T cells by disease type: Plot shows maximum (Max) distance (in μm) between CLEC9A^+^ DCs and TCF1^+^ or TCF1^–^ T cells in MM (*n* = 70), SMM (*n* = 12), or MGUS (*n* = 13). (**C**) Proximity of CLEC9A^+^ DCs and TCF1^+^ or TCF1^–^ T cells by disease risk. Plot shows maximum distance (in μm) between CLEC9A^+^ DCs and TCF1^+^ or TCF^–^ T cells in patients with HR MM (HR cytogenetics or PFS <2 years) versus DCs from non-HR patients. Standard risk (SR), *n* = 37; HR *n* = 31. Graphs in **B** and **C** show the maximum ± SEM. Each dot represents a unique patient or sample. *P < 0.05, **P < 0.01, ***P < 0.001, and ****P < 0.0001, by Brown-Forsythe and Welch’s ANOVA test with Dunnett’s T3 multiple-comparison test. (**D** and **E**) NanoString DSP analysis of CLEC9A^hi^ versus CLEC9A^none^ lesions. mIF on serial sections was performed to identify CLEC9A^hi^ (12 ROIs) versus CLEC9A^none^ (*n* = 24 ROIs). (**D**) Graph shows the top differentially enriched pathways between CLEC9A^hi^ and CLEC9A^none^ ROIs. (**E**) Volcano plot shows DEGs between CLEC9A^hi^ and CLEC9A^none^ ROIs.

As discussed earlier, regions of T cell enrichment in MM lesions were typically found to be in proximity to CLEC9A^+^ DCs. The phenotype of CLEC9A^+^ cells as being typical of conventional type 1 DCs and distinct from CD14^+^ myeloid cells was confirmed by mass cytometry (Supplemental Figure 16). We observed that CLEC9A^+^ cells were often typically located at the edge of tumor masses (Supplemental Figures 17 and 18). The proximity of CLEC9A^+^ DCs and T cells also created a visual impression of T cell gradients emanating from CLEC9A^+^ hotspots (Figure 4A). In order to quantify this, we analyzed differences in T cell density as a gradient based on proximity to CLEC9A^+^ DCs, versus the cross-gradient as a control. These analyses revealed a clear gradient with higher T cell density in the CLEC9A^+^ DC–proximal region(s) compared with DC-distal ones. This effect was not observed when T cell density was compared across the cross-gradient (Figure 4A). Gradients from CLEC9A^+^ DCs included several T cell subsets including CD4^+^, CD8^+^, CD3^+^TCF1^+^, and CD8^+^GZMB^+^ T cells, as well as NK cells, but not CD68^+^ myeloid cells, consistent with DCs being the nodes of immune activation (Supplemental Figure 19). Analysis of Ki67 on T cells as a proliferation marker also demonstrated a gradient from CLEC9A^+^ cells, consistent with in situ activation (Supplemental Figure 20). T cells proximal to CLEC9A^+^ DCs consisted of both TCF1^+^ and TCF1^–^ T cells. However, DC–TCF1^+^ T cell proximity, as measured by maximum CLEC9A-TCF1 distance, was further for MM biopsies relative to MGUS or SMM biopsies, consistent with closer DC–TCF1^+^ T cell proximity in MGUS (Figure 4B). As a control, these cohorts did not differ in terms of distances between CD68^+^ myeloid cells and TCF1^+^ or TCF1^–^ T cell subsets (Supplemental Figure 21). These effects were specific for the TCF1^+^ T cell subset, as distances between CLEC9A and TCF1^–^ T cells were not different in these cohorts (Figure 4B). Among patients with MM, the CLEC9A-TCF1 maximum distance also correlated with disease risk/outcome and was higher in patients with clinical high-risk (HR) disease (HR cytogenetics or progression-free survival [PFS] <2 years) (Figure 4C). These groups did not differ in terms of maximum distance between TCF1^–^CD3^+^ T cells and CLEC9A^+^ DCs. NanoString digital spatial profiling (DSP) analysis was used to evaluate differentially expressed genes (DEGs) enriched in CLEC9A^hi^ regions, which were identified through mIF staining of serial sections. These analyses revealed that CLEC9A^hi^ regions were enriched for several immune-related genes and pathways consistent with local immune activation (Figure 4, D and E). Together, these data suggest that CLEC9A^+^ DC regions may represent hotspots of local T cell activation and that the proximity of CLEC9A^+^ DC–TCF1^+^ T cell interaction may correlate with disease state and risk status.

### Spatial architecture and heterogeneity of myeloid compartment.

**Figure 5:**
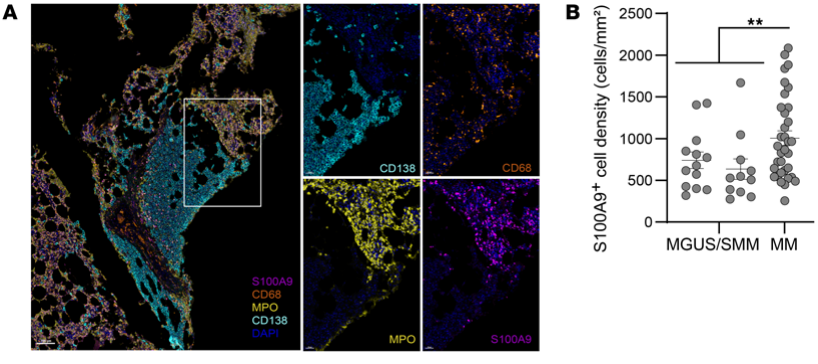
Spatial architecture of the myeloid compartment. mIF was performed on FFPE sections (see Methods and Supplemental Figure 2 for multiplex panel BM3 used for staining). (**A**) Representative IHC images show a pattern of staining for S100A9, MPO, CD68, and CD138 in MM bone marrow. Note that while CD68^+^ myeloid cells infiltrated CD138^+^ tumor clusters, S100A9^+^ myeloid and MPO^+^ myeloid/neutrophilic cells were predominantly located outside the tumor clusters (original magnification, ×8; insets, ×28.8). (**B**) S100A9^+^ cell density in MGUS, SMM, and MM. Bar graphs show the mean ± SEM. Each dot represents a unique patient/sample. MGUS *n* = 13; SMM *n* = 11; MM *n* = 34. **P < 0.01, by unpaired *t* test with Welch’s correction.

While CLEC9A^hi^ regions were enriched for immune pathways, CLEC9A^none^ regions were instead enriched for genes such as myeloperoxidase (MPO), S100A8, and S100A9 (Figure 4E), which have been previously implicated in myeloid or granulocytic suppressor cells (MDSCs) (16). In prior studies, we and others had also identified S100A8 and S100A9 as markers of myeloid cell populations with adverse features (10, 16). Therefore, we used mIF to characterize the spatial aspects of these cells (Supplemental Figure 2, panel BM3). We found that both S100A9^+^ myeloid cells and MPO^+^ myeloid/neutrophilic cells were abundantly present throughout MM marrows (Figure 5A and H&E-stained images in Supplemental Figure 6). Interestingly, both cell types were present predominantly in the nontumoral regions of the marrow, with little infiltration into dense tumor clusters themselves (Figure 5A and Supplemental Figure 22). In order to confirm that this finding was not due to antibody interference, the staining pattern was reverified with chromogenic assays (Supplemental Figure 23). Therefore, the pattern of infiltration of S100A9^+^ myeloid cells was quite distinct from that of CD68^+^ myeloid cells, as the latter readily infiltrated tumors (Figure 1). S100A9^+^ cell density was higher in MM relative to MGUS tumors (Figure 5B). Taken together, these data illustrate the complex spatial architecture of the myeloid compartment, with some cell types (e.g., CD68^+^ myeloid cells) consistent with tissue-resident cell populations that readily infiltrated tumors, while others (e.g., S100A9^+^ myeloid cells) were predominantly outside tumors and may reflect systemic myeloid dysregulation as an early event during malignant transition (16).

### Immune infiltration and clinical outcome in MM.

**Figure 6:**
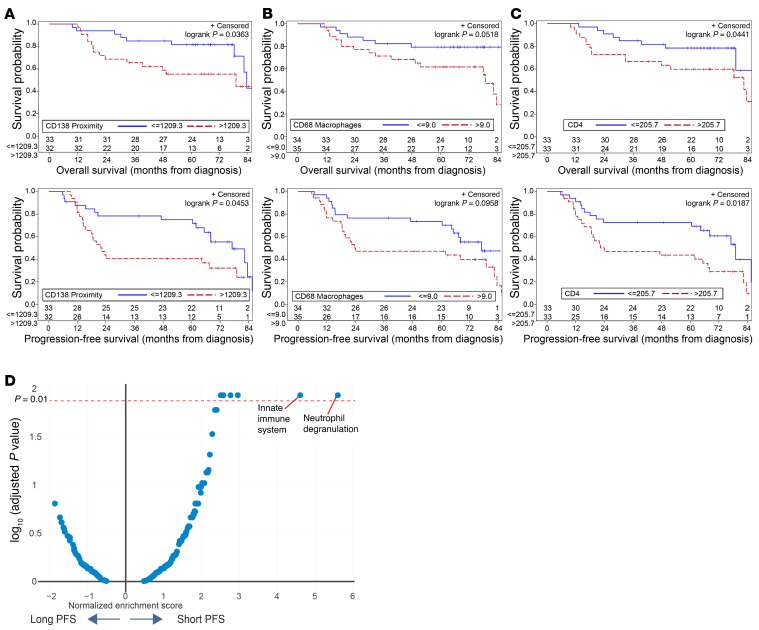
Immune infiltration and clinical outcomes in MM. (**A**–**C**) Kaplan-Meier plots showing OS and PFS in MM cohorts, split based on selected variables as in Table 1. Variables are split at the median for the cohorts. (**A**) CD138 proximity (based on the maximum number of CD138^+^ tumors cells within a 1,000 μm radius) and OS/PFS in the MM cohort. (**B**) CD68 expression and OS/PFS in the MM cohort. (**C**) CD4^+^ T cell density and OS/PFS in the MM cohort. (**D**) Pathways for DEGs in tumor-sparse regions by PFS: mIF was performed to first identify tumor-sparse ROIs. NanoString DSP analysis was performed to identify DEGs by PFS in tumor-sparse ROIs. Volcano plot shows differential pathways for DEGs in tumor-sparse ROIs between patients with short (<2 yr) (*n* = 11) or long (>5 yr) (*n* = 13) PFS.

Differences in immune infiltration and cell states between the MM and precursor states discussed earlier support the potential role of tumor-immune interactions in early myeloma. We used data for immune composition in terms of individual cell types as well as proximity analysis in the MM cohort to identify correlates of PFS and overall survival (OS) in MM biopsies. Of the variables tested, increased CD138 proximity (an indirect measure of cluster formation) correlated with both OS and PFS (Figure 6A and Table 1). In addition, an increase in CD68^+^ myeloid cells and CD4^+^ T cells approached significance for reduced PFS and OS, respectively, in a multivariable analysis (Figure 6, B and C, and Table 1). NanoString DSP was utilized as an orthogonal approach to validate these data and identify DEGs that correlated with extremes of outcome (PFS <2 years versus >5 years). Top genes in the initial comparisons were largely derived from tumors and included genes associated with HR genetics such as FGFR3 and MYC, which are linked to shorter PFS (17, 18), and genes associated with a differentiated PC phenotype (e.g., SLAMF7, IRF4) or standard-risk genetics (cyclin D1 [CCND1]), which are linked to longer PFS (Supplemental Figure 24). Therefore, we stained serial sections with mIF to first identify tumor-sparse regions of interest (ROIs) and compare DEGs and pathways in these ROIs in patients with disparate outcomes. These analyses revealed that the top pathways associated with short PFS were those associated with innate immunity, including granulopoiesis (Figure 6D).

## Discussion

**Figure 7:**
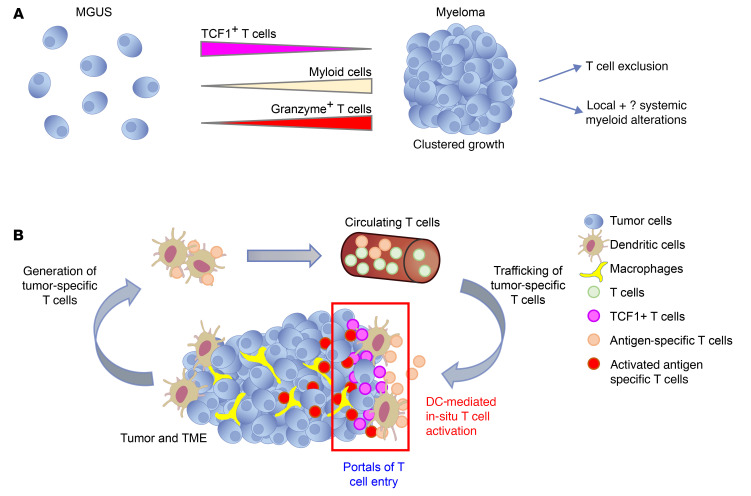
Proposed models. (**A**) Cluster formation and immune changes with malignant transition. Evolution from MGUS to MM is accompanied by a tumor-intrinsic capacity to form clusters. This transition is accompanied by loss of TCF1^+^ stem memory–like T cells, increased GZMB^+^ effector T cells, as well as alterations in the myeloid compartment. Clustered tumor growth sets the stage for T cell exclusion and spatial immune escape during malignant transition. (**B**) Role for DC-mediated antigen presentation in T cell entry. The cancer immunity cycle, as initially proposed, consisted of an afferent phase involving the generation of antigen-specific T cells by DCs and an efferent phase involving killing of tumor cells by tumor-specific T cells. Data in this study support an additional role for tumor-associated DCs (red boxed area), where the entry of antigen-specific T cells into tumor clusters depends on antigen-specific activation of T cells in situ by professional APCs. Hence, T cell infiltration into MM tumors is not random but occurs through portals of entry and antigen presentation hotspots containing antigen-presenting DCs. TCF1^+^ T cells are found in proximity to CLEC9A^+^ DCs, and the proximity of these cell types correlates with disease stage and risk.

These studies combined high-dimensional spatial profiling, machine learning, and in vitro/in vivo modeling to gain insights into spatial tumor-immune alterations in MM and its precursor MGUS. Spatial aspects of tumor-immune interactions have to date been extensively studied in malignant solid tumors, in which infiltration of T cells into tumors affects outcomes and the response to immune therapy (12). However, how these spatial interactions change in the context of hematologic malignancies and particularly with evolution from premalignancy in humans is not known. Our data identify several distinct alterations in spatial aspects of tumor/immune infiltration in the context of the transformation of MGUS to MM (Figure 7A). These studies complement prior studies of blood and bone marrow aspirates in MM, demonstrating that variance in immune composition affects the response to vaccines and therapy (19, 20). Clustered growth of tumor cells as a feature of MM, but not MGUS, creates the potential for immune exclusion as a possible mechanism underlying loss of immune surveillance in the transition of MGUS to MM and the possibility that malignant transition of MGUS may occur in spatially distinct regions that may be more protected from immune control. These data also suggest that entry of T cells into MM lesions is not random and is affected by at least 2 related factors: nature and the degree of costimulation and target recognition/antigen specificity, both provided in situ by antigen-presenting DCs, which may mark portals of entry. Synthetic bypass of this biology may therefore underlie the mechanism of action of chimeric antigen receptor–T cell (CAR-T) and bispecific therapies in MM (6). These data also suggest an important role for the CD2/CD58 axis, which is consistent with emerging data about this axis in lymphoma (21, 22). Engaging this pathway may therefore allow improvement of T cell redirection approaches in MM (6). Prior studies have suggested that the capacity to respond to agonistic signaling may be greater in the subset of TCF1^+^ stem-like memory T cells (23). Therefore, progressive attrition of these T cells, as recently described in MM (24), may eventually limit the capacity of T cells to mediate long-term control.

Harnessing immunity to neoantigens is an area of active research. However, mechanisms underlying the entry of antigen-specific T cells into human tumors remain poorly understood. The concept that entry of T cells is enhanced following antigen-specific stimulation by DCs in situ suggests a novel role for DC-mediated antigen presentation in the effector phase of the cancer immunity cycle, in addition to its well-studied role in the proximate arm, e.g., induction of tumor-specific T cells, both of which may be affected by immune checkpoints (25, 26) (Figure 7B). These data support a model in which DCs need to be in proximity to tumors in order to promote entry of antigen-specific T cells. Tumor-associated DCs may therefore serve as important portals for the entry of antigen-specific T cells into tumors. The concept that CLEC9A^+^ conventional type1 DCs (cDC1s) may serve as APCs for activation of T cells in situ in MM is also consistent with their role as a DC subset specialized for cross-presentation (27) and with the data correlating these cells with response to immune therapies in solid tumors (28, 29). The nature of tumor-infiltrating APCs may also affect the properties of T cells in tumors, as indicated by the close interactions between CLEC9A^+^ DCs and TCF1^+^ T cells that we observed. Strategies to enhance CLEC9A^+^ cDC1s within the TME may therefore enhance the durability of immune therapies in MM. Infiltration of cDC1s was recently also linked to the durability of responses following B cell maturation antigen–CART-T (BCMA–CAR-T) therapy in MM (20).

Our findings also reveal the spatial complexity of myeloid/DC architecture in MM, with some myeloid cell subsets (e.g., cells expressing S100A9) that diffusely infiltrated the marrow but resided predominantly outside tumor lesions. These subsets carry many of the markers typically associated with MDSCs (10, 16) and have been characterized as such in the circulation (16), consistent with the possibility that immune suppression mediated by such cells is systemic and extends beyond the immediate TME (30). Besides myeloid cells, CD4^+^ T cells correlated with worse outcomes in MM, in accordance with other studies (31), and the putative role of CD4^+^ T cell subsets in MM bone disease (e.g., Th17 cells) (32) and in providing help to B cells (33).

In addition to changes in immune cells, the pattern of tumor infiltration, such as clustered growth, may also be clinically relevant. Our finding that this pattern was reproduced in humanized mice suggests that this is a tumor-intrinsic feature of MM. Further studies are needed to better understand this biology, which may also be occurring in the murine Vkappa-MYC model (Vκ*MYC) (34). Machine-learning approaches such as those utilized here may, however, be useful to quantify this biology for clinical benefit. For example, cluster formation (quantified by PC proximity) may be the MM histologic equivalent of collective invasion/tissue infiltration, which is commonly used for pathologic diagnosis of infiltrating carcinoma (35). Application of such tools may therefore provide a much-needed pathologic marker for the malignant phenotype and help identify patients with malignant potential in intermediate lesions such as SMM and reduce the current reliance on end-organ damage to define the malignant phenotype in MM. We found that CD138 proximity as a quantitative measure of clustered tumor growth was also correlated with outcome in patients with MM. As tumor clusters set the stage for immune exclusion, these findings further illustrate the potential clinical importance of spatial immunology in MM.

The strengths of this study are the use of several complementary and orthogonal approaches, including both in vitro and in vivo models and testing tissues from defined cohorts of patients at initial diagnosis. Importantly, spatial analyses in this study were based on whole slides, in contrast to approaches that often do not capture the entire tissue biopsy. This issue is particularly relevant in view of the spatial and regional heterogeneity within a single biopsy that we observed. One limitation is that bone marrow biopsies were based on the current clinical standard of care in MM, which does not involve imaging-guided biopsies. To our knowledge, while this is the largest MM cohort analyzed to date for spatial immunology, further studies are needed to evaluate the effect of tumor genetics on this biology. Insights into the regulation of entry of antigen-specific T cells described here also have broad implications for improving T cell redirection, as well as for emerging strategies targeting antigen-specific T cells in MM.

## Methods

### Human cell lines and primary samples from patients.

The human MM cell lines U266, XG-1, KMS-18, KMS-12-BM, and INA-6 were maintained in complete RPMI-1640 media supplemented with 20% heat-inactivated FBS (Gibco, Thermo Fisher Scientific), antibiotics (penicillin-streptomycin, solution, MilliporeSigma), 2.0 mM l-glutamine (Gibco, Thermo Fisher Scientific), and 1.0 mM sodium pyruvate (Gibco, Thermo Fisher Scientific) as previously described with some minimal modifications (36). Growth media for INA-6 were supplemented with recombinant human IL-6 (rhIL-6) at a final concentration of 5 ng/mL (R&D Systems). Parental U266, XG-1, KMS-18, KMS-12-BM, and U266-Cas 9 cell lines were a gift of Lawrence H. Boise (Emory University, Atlanta, Georgia, USA). The INA-6 cell line was obtained from Leibniz Institute DSMZ-German Collection of Microorganisms and Cell Culture GmbH (DSMZ no. ACC 86z). Blood and bone marrow specimens from patients with monoclonal gammopathies (MM or MGUS) were collected. Buffy coats purchased from New York Blood Center or LifeSouth were used as a source of T cells from healthy donors. For in vitro clonogenic assays, tumor cells (CD138^+^) from primary bone marrow mononuclear cells (BMMNCs) were enriched using CD138 magnetic selection (Miltenyi Biotec). Isolated CD138^+^ cells were used to generate colonies in the presence of 5 ng/mL rhIL-6. To generate cell lines expressing model neoantigen, HLA-A2^+^ parental U266 or XG-1 cell lines were first transduced with the pCMJJ4-CMV-Flu pep-IRES-Thy1.1 vector to generate U266-MP or XG-1-MP cells. Furthermore, U266-MP cells were transduced with the pLL-CMV-rLuc-T2A-GFP-mPGK-puromycin lentivector (System Biosciences, catalog LL310VA-1), following the manufacturer’s protocol, to generate U266-MP-rLuc cells.

### Biopsy materials.

Bone marrow biopsy materials for study were selected from archival formaldehyde-fixed, paraffin-embedded (FFPE) trephine biopsies from patients with plasma dyscrasias. FFPE blocks were sectioned at 4 μm, and the sections were placed on positively charged slides. H&E staining was performed and assessed for adequate tissue and cellularity. Once multiplex immunofluorescence staining was done, images were evaluated for adequate DAPI staining and excessive background. The samples selected had consistent DAPI staining over the majority of the tissue. Unstained slides were stored at –20°C.

### Development of mIF panels.

The optimal concentration of individual antibodies under consideration for panels was initially analyzed on control bone marrow biopsy slides. Once the appropriate antibody concentration was determined, position staining was performed to ascertain optimal staining positions for each antibody. IHC was achieved utilizing a Ventana DISCOVERY ULTRA system (Roche) autostainer and reagents. Opal (Akoya Biosystems) tyramide signal amplification fluorophores were chosen on the basis of expected marker abundance and cellular localization of markers. Prospective panels were then created and validated. Validation involved comparison of standard chromogenic staining to single-color Opal staining in the panel-specific order to verify a similar staining pattern and dropout controls to verify no changes in staining pattern when stained in the full panel.

### Reagent preparation.

Adequate quantities of all primary antibody dilutions for each panel were made in Diamond Antibody Diluent (Cell Marque, 938B-09) prior to staining. All Opal fluorophores for each panel were reconstituted in DMSO, pooled, and then aliquoted for batch use to avoid variability and freeze-thaw issues. Opal fluorophore aliquots were diluted in 1× Plus Amplification Diluent (Akoya Biosystems) just prior to staining.

### Multiplex staining.

Final staining protocols (Supplemental Figure 2) included initial steps of baking and deparaffinization with EZ-Prep solution (Roche), heat-induced epitope retrieval (HIER) with Cell Conditioning 1 (CC1, Roche), and application of DISCOVERY Inhibitor (Roche) to block endogenous peroxidase activity, all performed on the DISCOVERY ULTRA autostainer. Staining was accomplished by an iterative process of incubations with primary antibodies, OmniMap HRP secondary antibodies (Roche) corresponding to the species of the primary antibody, and application of the Opal (Akoya Biosystems) tyramide signal amplification fluorophores followed by a denaturation cycle using high heat (93°C) and Cell Conditioner 2 (CC2, Roche). Slides were counterstained for nuclei with Spectral DAPI (Akoya Biosystems). Upon completion of the staining process, slides were removed from the autostainer, briefly soaked in a detergent solution to remove liquid coverslip residue, and then cover-slipped using Vectashield Antifade mounting medium (Vector Laboratories). Slides were allowed to cure for 24 hours in the dark and then stored at 4°C prior to imaging.

### Image acquisition.

A Vectra Polaris Automated Quantitative Pathology Imaging System was used to acquire whole-slide multispectral images. Ideal exposures were determined by averaging the exposures generated by the Vectra Polaris software across a range of slides.

### Annotation, unmixing, and analysis.

Once whole-slide images were acquired, manual assessment of the images was done to verify the quality of staining. Images deemed appropriate for analysis displayed sufficient cellularity, adequate DAPI signal for cell segmentation, and a lack of excessive background that would hinder cell phenotyping. Images were then annotated by selecting areas for analysis. To create analysis algorithms, 1 to 3 areas were “stamped” for “inForm projects” across many slides to provide a representative sampling of the staining results.

For each separate panel, adaptive cell segmentation was done on the basis of DAPI nuclear staining and select cell membrane markers. Phenotyping was performed utilizing a layered approach, with each phenotype trained separately. Briefly, representative positive and negative cells for each marker were selected across the training images. “Train classifier” was then selected, which marks all cells as either positive or negative for the marker being trained. Visual analysis of the training results was done, allowing for correction of errantly identified cells and further training. This process was repeated for each marker separately.

Whole-slide image analysis was performed for all samples using “Batch Analysis,” avoiding large areas of tissue folding, bubbles, bone, fat, or other areas lacking cells. The maximum possible number of stamps or ROIs for “inForm batch” were selected for each image. Images were analyzed in batches of approximately 10 for each phenotype. As phenotypes were gathered for each batch, the data for each phenotype were merged. Further data processing was accomplished using Phenoptr and Phenoptr Reports and R Studio add-ins from Akoya Biosystems. Merged files were then consolidated, bringing all phenotypes for each batch together into a text file, in which each line included all attributes of a single cell as well as the X and Y coordinates for that cell. This consolidated file was further analyzed, allowing for identification of specific combinations of phenotypes and calculation of cell counts, cell percentages, cell densities, expression levels, nearest neighbors, and the counts of cells within a specified distance. CD138 cell proximity analysis was performed using custom Python code to query the counts within the file to quantify the number of CD138 cells within a specified radius of a CD138 cell. To evaluate T cell gradients from DCs, areas visually identified as having CLEC9A^+^ DCs were manually segmented into 3 roughly equal sections, with a section proximal to the DCs and 2 other sections progressively distal to DCs, while utilizing cross-gradients as controls. Batch analysis of the tissue was performed with the same algorithms used for whole-tissue analysis. T cell density was evaluated for each region. For some images, additional analysis was performed using HALO software from Indica Labs according to the manufacturer’s guidelines.

### In vitro modeling of human T cell infiltration into MM colonies.

Human T cells were isolated using the Pan T Cell Human kit (Miltenyi Biotec) and labeled (if needed) with either PKH26 red cell fluorescent linker or PKH67 green cell fluorescent linker. For in vitro stimulation, approximately 1 × 10^6^ isolated T cells were stimulated by different methods such as the use of soluble α-CD3/CD28/CD2 or α-CD3/CD28 human ImmunoCult activators (STEMCELL Technologies); α-CD3 (clone HIT3a) (BioLegend); α-CD28 (clone CD28.2); α-CD2 (clone TS1/8); and α-CD2/CD28 (BioLegend) according to the manufacturers’ recommendations. For some experiments, naive T cells were isolated using the Naive Pan T Cell Isolation kit (Miltenyi Biotec). Memory T cells were isolated using the Memory CD8^+^ and Memory CD4^+^ Human T Cell Isolation kits (Miltenyi biotec).

Human MM cell lines (U266, XG-1, KMS-18, KMS-12-BM and INA-6) were plated in methylcellulose media (H4434 classic media, STEMCELL Technologies) according to the manufacturer’s recommendations and using protocols adapted from published reports (36, 37). For some experiments, U266 MM cells were infected with inactivated influenza virus (Charles River Laboratories, catalog 10100782) prior to clonogenic assays. For some experiments, CD138^+^ cells from MM bone marrow were isolated with magnetic selection (Miltenyi Biotec) according to the manufacturer’s recommendations. MM tumor colonies were detected 14 days after plating the cells in suspension in methylcellulose media. To allow the formation of INA-6 colonies or primary MM colonies, growth media were supplemented with rhIL-6 (5 ng/mL). Once the colonies were established, they were marked using an inverted light microscope, and then fluorescence-labeled unstimulated or stimulated 5,000–30,000 human T cells were delivered mechanically near the colonies using a micropipette. For some experiments, these cocultures were incubated for 24 hours at 37°C and 5% CO_2_ to allow the interaction between target colonies and effector T cells. T cell infiltration of myeloma colonies was assessed using a wide-field Olympus IX71 microscope and a 20× objective to capture the images. Confocal images were taken with the 40× oil objective and a white laser (470–670 nm) at different time points. Hybrid detectors collected the fluorescent signal of PKH67, a green fluorescent cell linker (490–502 nm), and PKH26 red fluorescent cell linker (551–567 nm). The degree of T cell infiltration (area of fluorescent T cells within the myeloma colonies) was quantified using Fiji software.

### Antibody or CRISPR-mediated inhibition of CD2-CD58 interactions.

To block CD2-CD58 interactions, a purified monoclonal antibody against human CD58 was used (clone TS2/9, BioLegend). The α-CD58 antibody or the isotype control was added to the colonies for 2 hours at 37^o^C, 5% CO_2_. Then, colonies were cocultured with unstimulated or α-CD3/CD28/CD2–stimulated T cells for 24 hours. T cell infiltration levels were assessed using a fluorescence microscope. For some experiments, CD58 was deleted in Cas9-expressing U266 cells (gift from L. Boise, Emory University, Atlanta, Georgia, USA) using CRISPR following electroporation of sgRNA (sequence 5′ to 3′: UGG UUGCUGGGAGCGACGCG, Synthego) using the Amaxa 4D electroporator (Lonza). Knockdown of CD58 was confirmed by flow cytometry.

### DC-mediated generation of antigen-specific T cells.

Monocyte-derived DCs (mo-DCs) were generated from purified CD14^+^ monocytes in the presence of IL-4 (25 ng/mL; R&D Systems) and GM-CSF (20 ng/mL; sagramostim [LEUKINE], Genzyme) and matured with LPS (25 ng/mL; MilliporeSigma) as previously described (26). For antigen-specific T cell stimulation, mature mo-DCs from HLA-A2^+^ donors were pulsed with 1 μg/mL HLA A2–restricted influenza matrix peptide (Flu-MP) (sequence GILGFVFTL, AnaSpec, catalog AS-28310) and cocultured with autologous T cells at a DC/T cell ratio of 1:20 in the presence of IL-2 (20 U/mL). After 1 week, T cells were restimulated with additional Flu-MP–loaded DCs in the presence of IL-2 (20 U/mL), IL-7 (5 U/mL), and IL-15 (5 U/mL). Flu-MP–specific T cells were identified by MHC tetramers (iTag Tetramer HLA-A2 Influenza-M1 [GILGFVFTL], MLB International) and sorted after 2 weeks of culturing (26). Fresh propidium iodide (PI) dye (10 μg) (Invitrogen, Thermo Fisher Scientific) was used in some experiments to monitor tumor cell lysis. For experiments relating to entry of antigen-specific T cells, antigen-loaded (or unpulsed) DCs were added to tumor colonies 4 hours prior to the addition of T cells. For some experiments, DCs were preincubated with 50 μg/mL α–MHC-1 (clone W6/32, BioLegend) or an isotype control for 1 hour at 37°C prior to peptide loading.

### In vivo modeling in MISTRG6 mice.

Eight- to 10-week-old MIS^h/m^TRG6 male or female mice (9) were irradiated with 1.7 Gy, and 4 hours later, irradiated mice were injected intrafemorally with approximately 2 × 10^6^ to 4 × 10^6^ primary MM or MGUS bone marrow cells, as described previously (9). Prior to injection, primary MM or MGUS BMMNCs were depleted of T cells. Engraftment was monitored by ELISA, which was performed on mouse sera to detect human Igs as previously described (9). Mice were euthanized upon evidence of engraftment, and whole-bone mounts of murine bones (38, 39) were analyzed by IHC to evaluate the pattern of marrow involvement.

### Adoptive transfer of antigen-specific T cells.

In order to study the effects of adoptive transfer of antigen-specific T cells, MIS^h/m^TRG6 mice were first engrafted intrafemorally with 3 million U266-MP-rluc cells alone or with 5 × 10^5^ mo-DCs from A2^+^ donors pulsed with MP peptide. Engraftment was monitored using an in vivo imaging system (IVIS). On day 4 after cancer cell implantation, mice were then injected via retroorbital injection with 4 million T cells from the same donor, which had been previously expanded with MP-pulsed DCs. Mice were euthanized 7 days after T cell transfer, and the presence of T cells and tumor cells was analyzed by flow cytometry and IHC, as described above.

### GeoMx DSP.

Ten FFPE tissue samples from patients with MM were processed following the GeoMx DSP Slide Prep user’s manual (MAN-10087-04). The slides were baked at 60°C for at least 3 hours and then deparaffinized through Leica Biosystems BOND RX. Following proteinase K digestion, the Cancer Transcriptase Atlas (CTA) probe mix was added to the tissue for overnight hybridization. After that, the slides were washed with buffer and stained with CD138 (BioLegend, 356526), CD3 (Origene, AC210503), and Syto83 (Thermo Fisher Scientific, S11364) for 2 hours and then loaded onto the GeoMx DSP machine to scan 20× fluorescence images. ROI tissue samples were placed and collected into a 96-well plate. Oligonucleotides from each ROI were uniquely indexed using Illumina’s i5 × i7 dual-indexing system. After purification of the libraries, the samples were processed through Illumina’s NovaSeq. Fastq files were further processed using the GeoMx next-generation sequencing pipeline, and raw and Q3 normalized counts of all CTA targets in each ROI were obtained through NanoString GeoMx Data analysis software.

After removing the technical outliers, GeoMx DSP counts from each ROI were scaled to the 75th percentile of expression. The ROIs were categorized according to cell enrichment group. For this, serial sections of tissue were stained using multiplex IHC, and cell types were quantified within each DSP ROI. This information was then used to classify the DSP ROIs on the basis of the presence or absence of CLEC9A DCs as well as the density of CD138^+^ tumor cells. Within each group, differential expressions of genes was analyzed using a linear mixed model with a Benjamini-Hochberg–adjusted FDR. Genes with a FDR of less than 0.05 were considered to be significantly expressed and were used for the following pathway-based functional analysis. Gene set enrichment analysis (GSEA) was performed, and the normalized enrichment score (NES) was calculated using the gene set signatures from MsigDB and Reactome database.

### Statistics.

Statistical analysis was performed using GraphPad Prism version 9 (GraphPad Software), SPSS package version 27.0, and SAS version 9.4 (SAS Institute). Patient data were obtained from the myeloma database, which captures patient demographics and outcomes data and is continuously updated with periodic quality checks. Survival distributions were estimated using the Kaplan-Meier method and were compared using log-rank tests. Cox proportional hazards models were used to identify correlates of survival. Variables with a *P* value of less than 0.1 on univariate analysis were considered for inclusion in the final multivariable analysis. All statistical tests were 2 sided unless otherwise noted, and statistical significance was assessed at the 0.05 level.

### Study approval.

All studies involving human biospecimens were conducted following approval by the IRB of Emory University. Biospecimens were obtained following informed consent in accordance with the Declaration of Helsinki. All animal studies were conducted in accordance with protocols reviewed and approved by the IACUC of Emory University.

### Data availability.

The analytic code for mIF analysis can be downloaded from https://github.com/DhodapkarLab/mIF Values for patient-level data are reported in the Supporting Data Values file.

## Author contributions

MHR and NYV share first authorship, and the order of their names was determined alphabetically. KMD and MVD conceptualized the study, provided resources, conducted formal analysis, supervised the study, acquired funding, wrote the original draft of the manuscript, were responsible for project administration, and interpreted and analyzed the data. MHR designed and performed experiments including IHC analyses and in vivo studies, wrote the manuscript, analyzed and interpreted data, and reviewed and edited the manuscript. NYV designed and performed experiments including T cell infiltration and in vivo studies, wrote the manuscript, analyzed and interpreted data, and reviewed and edited the manuscript. DLJ oversaw specimen collection, performed data analysis and interpretation, and reviewed and edited the manuscript. AKN oversaw clinical data, data analysis, and interpretation and reviewed and edited the manuscript. AD, SSM, JM, SB, SP, MIA, and AAH assisted with in vitro and in vivo studies, performed data analysis and interpretation, and reviewed and edited the manuscript. GHS coordinated specimen collection and reviewed and edited the manuscript. JG and YL performed NanoString analysis and reviewed and the edited manuscript. LHB and VAG assisted with CRISPR studies and reviewed and edited the manuscript. VCP assisted with IHC studies, performed data analysis and interpretation, and reviewed and edited the manuscript. JMS conducted biostatistical analyses and data analysis and interpretation and reviewed and edited the manuscript. JLK, CCH, NSJ, and SL were responsible for the clinical aspects of the study, performed data analysis and interpretation, and reviewed and edited the manuscript.

## Supplementary Material

Supplemental data

Supplemental table 1

Supporting data values

## Figures and Tables

**Table 1:**
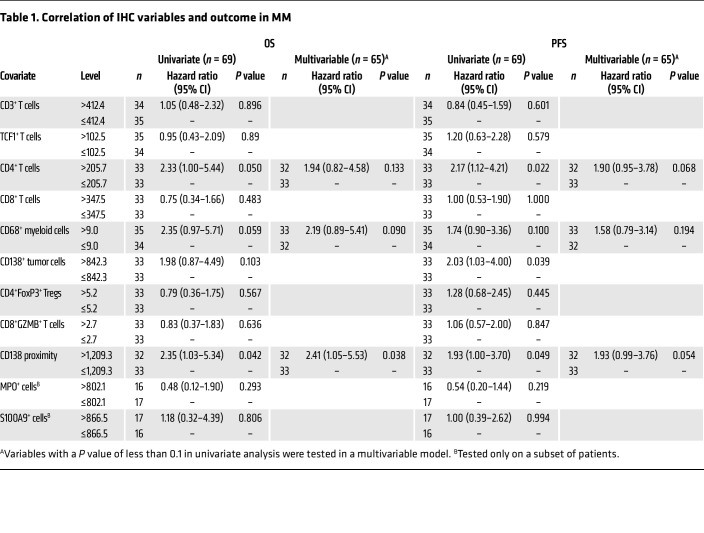
Correlation of IHC variables and outcome in MM

## Version 1 08/01/2023

Electronic publication
