# Supplementary material for: Regulation of antigen-specific T cell infiltration and spatial architecture in multiple myeloma and premalignancy
Source: J Clin Invest. 2023 Aug 1;133(15):e167629. doi: 10.1172/JCI167629 (PMC10378152; doi:10.1172/JCI167629)
Supplement: Supplemental data [file jci-133-167629-s009.pdf]

Supplemental Figure 1

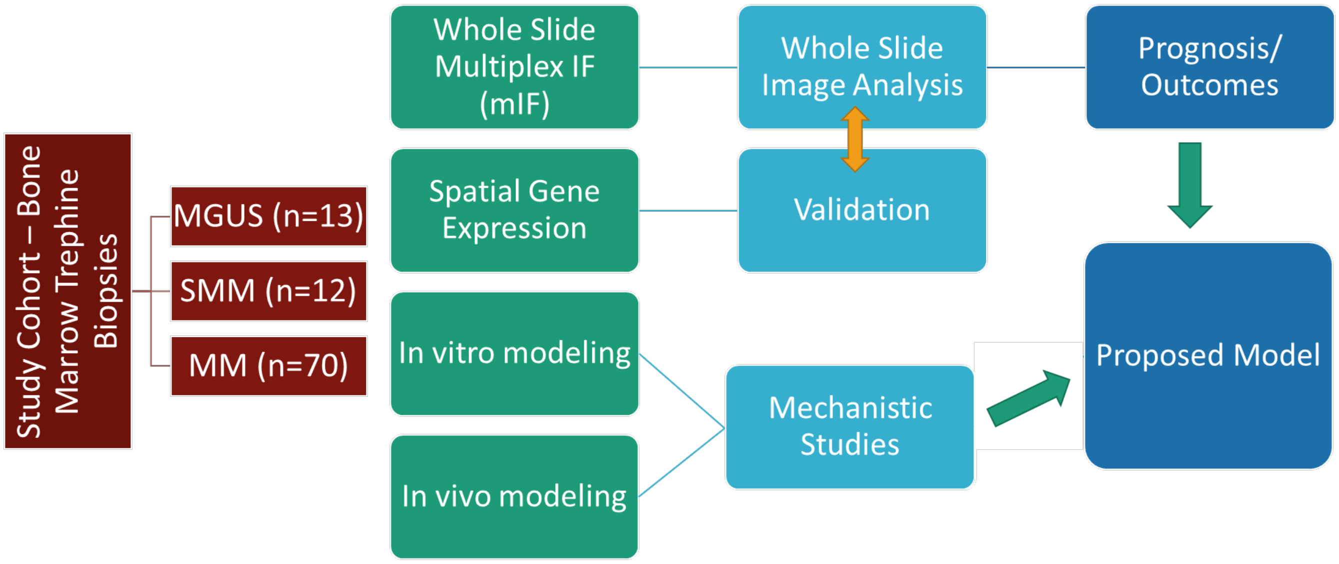


**Supplemental Figure 1. Overall approach.**

These studies utilize specimens from cohorts with MGUS, SMM and newly diagnosed MM and evaluate spatial changes utilizing multiplex IF (mIF), along with analyses of spatial gene expression. These data are integrated with findings from in vitro and in vivo models to gain mechanistic insights. These data are also utilized to identify correlates of outcome and develop a model for spatial regulation of tumors and immune cells in MM.

Supplemental Figure 2

**Panel: BM1**

| Order | Antibody | Clone | Vendor | Species | Antibody Dilution Factor | Incubation time (minutes) | Opal | Opal Dilution |
|---|---|---|---|---|---|---|---|---|
| 1 | TCF1 | C63D9 | Cell Signaling | Rabbit | 1:100 | 40 | 520 | 1:50 |
| 2 | CD68 | PG-M1 | Dako (Agilent) | Mouse | 1:200 | 44 | 620 | 1:50 |
| 3 | CLEC9A | EPR22324 | Abcam | Rabbit | 1:500 | 40 | 570 | 1:50 |
| 4 | CD138 | MI15 | Invitrogen | Mouse | 1:20 | 40 | 480 | 1:150 |
| 5 | CD3 | SP7 | Abcam | Rabbit | 1:100 | 44 | 690 | 1:50 |
| 6 | DAPI | | | | | 16 | | |

**Panel: BM2**

| Order | Antibody | Clone | Vendor | Species | Antibody Dilution Factor | Incubation time (minutes) | Opal | Opal Dilution |
|---|---|---|---|---|---|---|---|---|
| 1 | FOXP3 | 236A/E7 | Abcam | Mouse | 1:100 | 40 | 520 | 1:50 |
| 2 | Granzyme B | EP230 | CellMarque (Sigma) | Rabbit | 1:100 | 40 | 620 | 1:50 |
| 3 | CD8 | 144B | Abcam | Mouse | 1:40 | 40 | 690 | 1:50 |
| 4 | CD4 | EP204 | CellMarque (Sigma) | Rabbit | 1:50 | 120 | 570 | 1:50 |
| 5 | CD138 | MI15 | Invitrogen | Mouse | 1:20 | 40 | 480 | 1:150 |
| 6 | DAPI | | | | | 16 | | |

**Panel: BM3**

| Order | Antibody | Clone | Vendor | Species | Antibody Dilution Factor | Incubation time (minutes) | Opal | Opal Dilution |
|---|---|---|---|---|---|---|---|---|
| 1 | S100A9 | polyclonal | Abnova | Goat | 1:1000 | 40 | 520 | 1:50 |
| 2 | CD68 | PG-M1 | Dako (Agilent) | Mouse | 1:200 | 44 | 620 | 1:50 |
| 3 | CD3 | SP7 | Abcam | Rabbit | 1:100 | 44 | 690 | 1:50 |
| 4 | CD138 | MI15 | Invitrogen | Mouse | 1:20 | 40 | 480 | 1:150 |
| 5 | MPO | polyclonal | Agilent | Rabbit | 1:10,000 | 40 | 570 | 1:50 |
| 6 | DAPI | | | | | 16 | | |

**Supplemental Figure 2. Staining panels for mIF.**

The entire sample set was stained with panels BM1 and BM2. A subset of samples was also stained with BM3. Panels show details of staining protocols. Additionally, for a few cases a modified BM1 panel was used but with Ki-67 antibody (Clone MIB-1 from Agilent; Part Number: M724029-2) in place of CD68.

Supplemental Figure 3

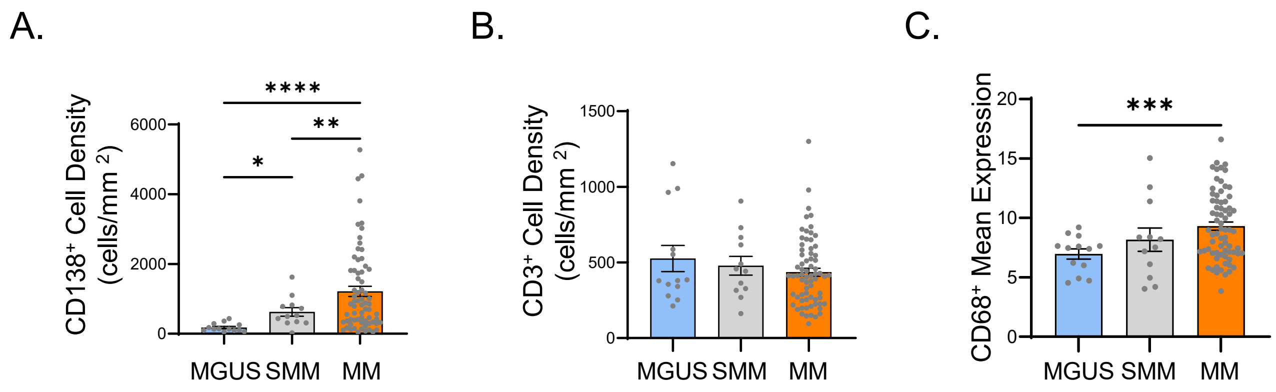


**Supplemental Figure 3. Immune composition of biopsy in MM, MGUS, and SMM.**

A. CD138⁺ cell density in the cohorts of MGUS (*n* = 13), SMM (*n* = 12) and newly diagnosed MM (*n* = 70). B. CD3⁺ T cell density in cohorts in 3A. C. CD68⁺ (mean expression) in cohorts in 3A. Bar graphs show mean ± SEM. Each dot represents a unique patient/sample. Brown-Forsythe and Welch ANOVA tests with Dunnett's T3 multiple comparisons test. *p<0.05, **p<0.01, ***p<0.001, ****p<0.0001

Supplemental Figure 4

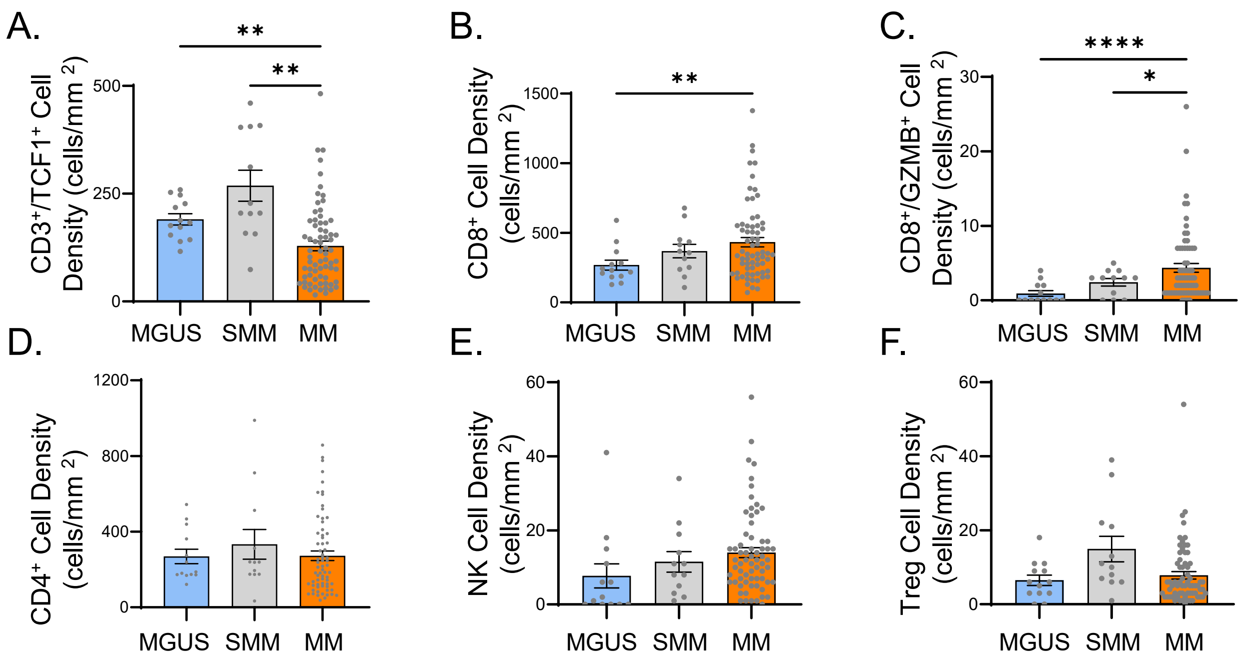


**Supplemental Figure 4. Composition of T and NK cells in MM, SMM and MGUS cohorts.**

Whole slide images of mIF analysis of diagnostic bone marrow from patients with MGUS (*n* = 13), SMM (*n* = 12) and MM (*n* = 70) were analyzed using machine learning and inForm$^{TM}$ software to identify cells and perform density analysis. 4A. $CD3^+/TCF1^+$ T cell density between MGUS, SMM and MM cohorts. 4B. $CD8^+$ T cell density between MGUS, SMM and MM cohorts. 4C. $CD8^+/GZMB^+$ T cells MGUS, SMM and MM cohorts. 4D. $CD4^+$ T cell density MGUS, SMM and MM cohorts. 4E. NK cells ($CD4^-/CD8^-/GZMB^+$) in MGUS, SMM and MM cohorts. 4F. $CD4^+/FOXP3^+$ Tregs in MGUS, SMM and MM cohorts. Bar graphs show mean ± SEM. Each dot represents a unique patient/sample. Brown-Forsythe and Welch ANOVA tests with Dunnett's T3 multiple comparisons test. *p<0.05, **p<0.01, ****p<0.0001

Supplemental Figure 5

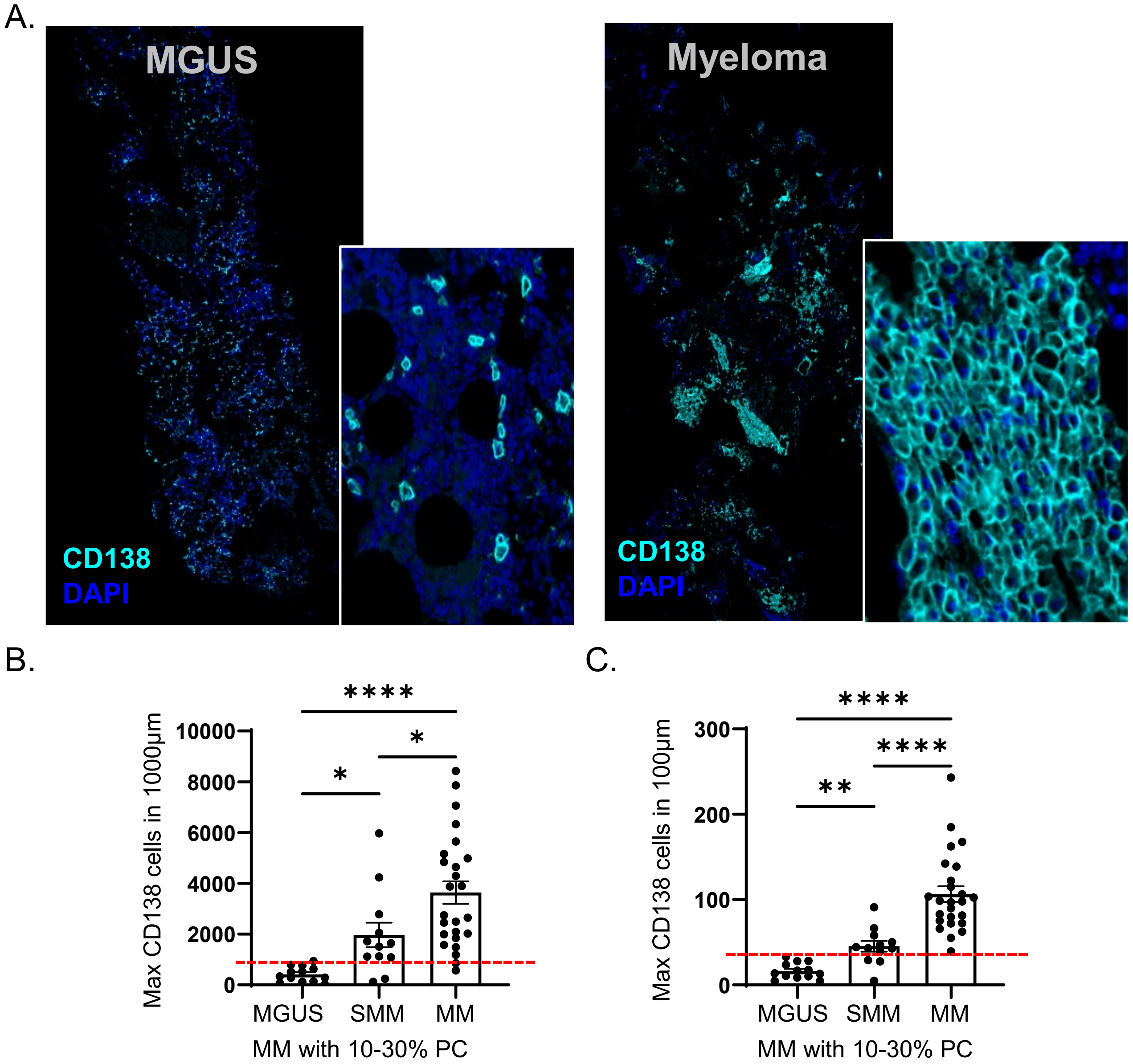


**Supplemental Figure 5. Clustered growth pattern in MM, MGUS, and SMM and CD138 proximity analysis.**

A. IHC of whole biopsy showing clustered growth of tumor cells in MM (1.25x, inset 40x), but not in MGUS. B. CD138 proximity analysis (1000 µm radius). C. CD138 proximity analysis (100 µm radius). Red line represents mean + 2 SD of MGUS. Bar graphs show max ± SEM. Each dot represents a unique patient/sample (MGUS *n* = 13, SMM *n* = 12 and MM *n* = 25). Brown-Forsythe and Welch ANOVA tests with Dunnett's T3 multiple comparisons test. *p<0.05, **p<0.01, ****p<0.0001

Supplemental Figure 6

Corresponds to Figure 1A (6.7x)

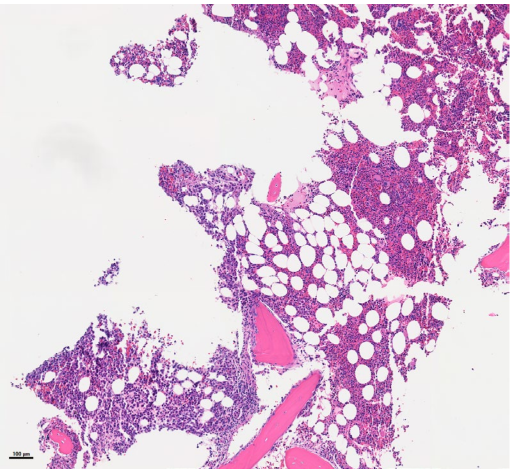

Corresponds to Figure 1B (20x)

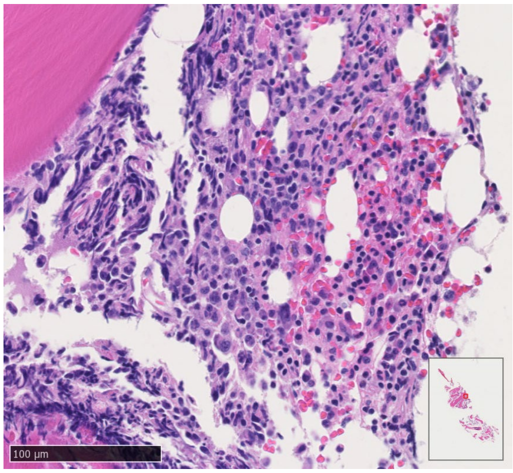


Corresponds to Figure 1C (2.5x, insets 15.7x)

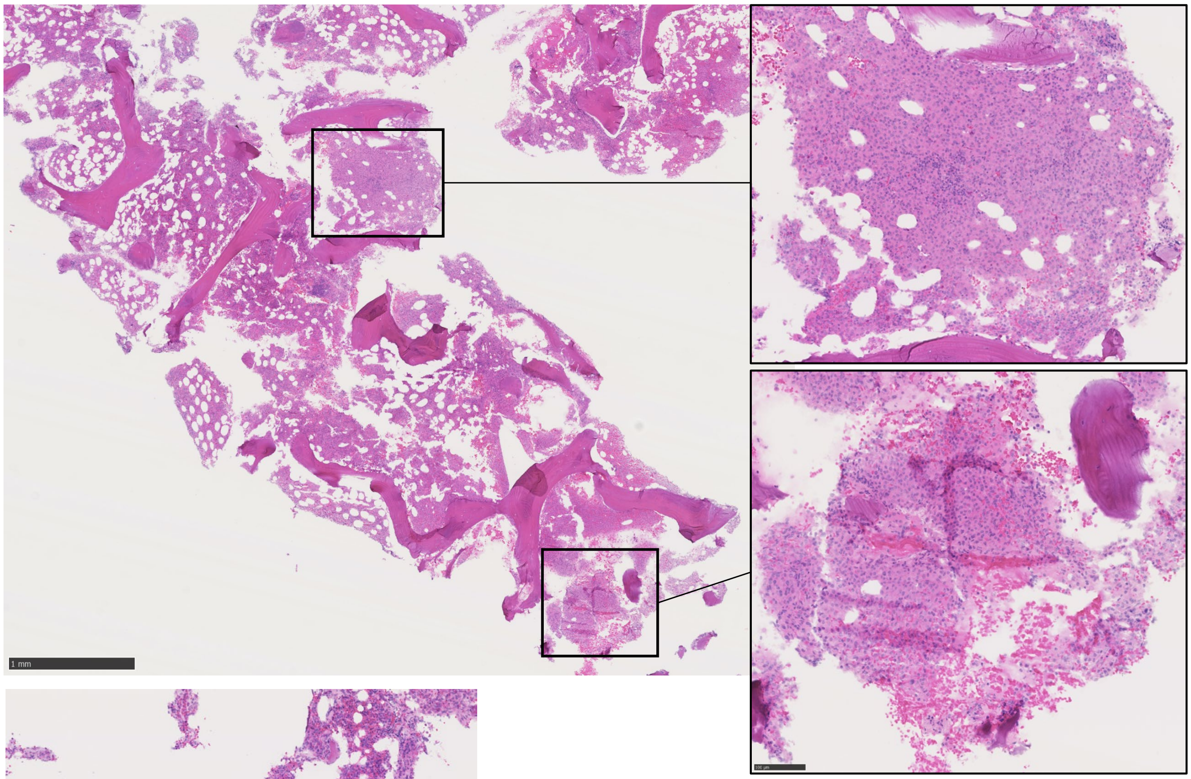


Corresponds to Figure 5 (8x, inset 20x)

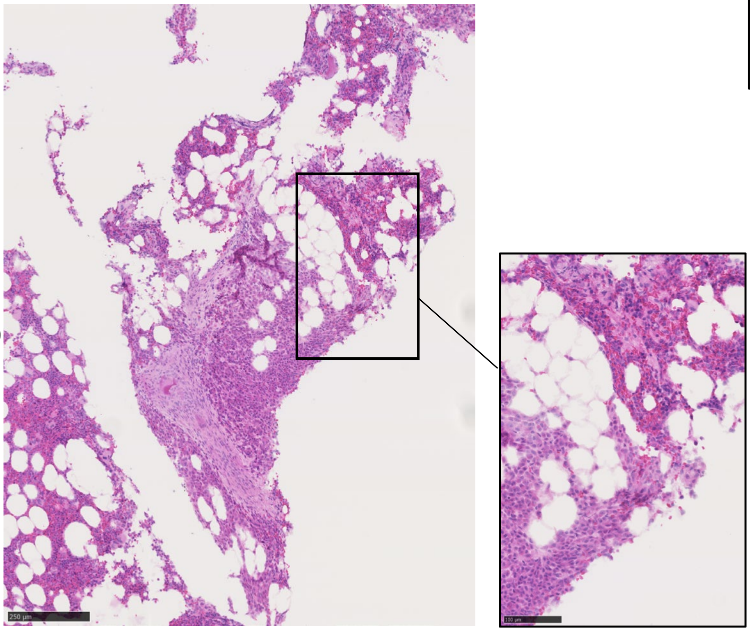

**Supplemental Figure 6**

Images of Hemotoxylin and Eosin (H&E) staining of tissues shown for mIF staining in Figures 1 and 5.

Supplemental Figure 7

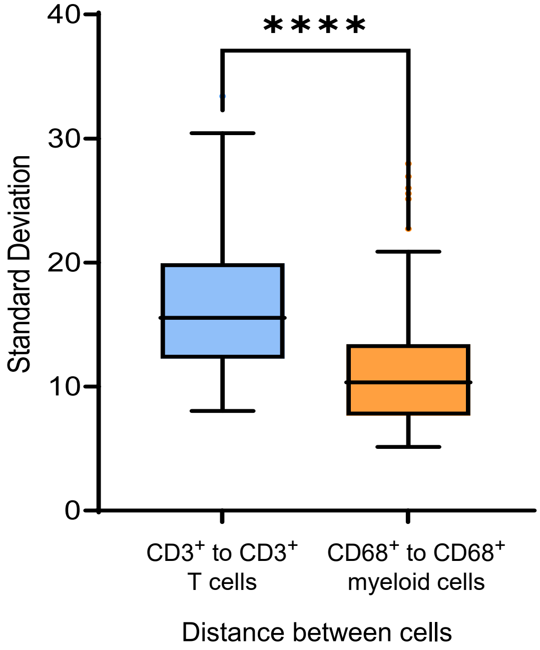


**Supplemental Figure 7.Variance in intercellular distance for CD3+ T cells versus CD68+ myeloid cells.**

Standard deviation of nearest neighbor distance for CD3 to CD3 T cells or CD68 to CD68 myeloid cells in MM patient biopsies (*n* = 70). Two-tailed unpaired t test with Welch's correction, ****p<0.0001

Supplemental Figure 8

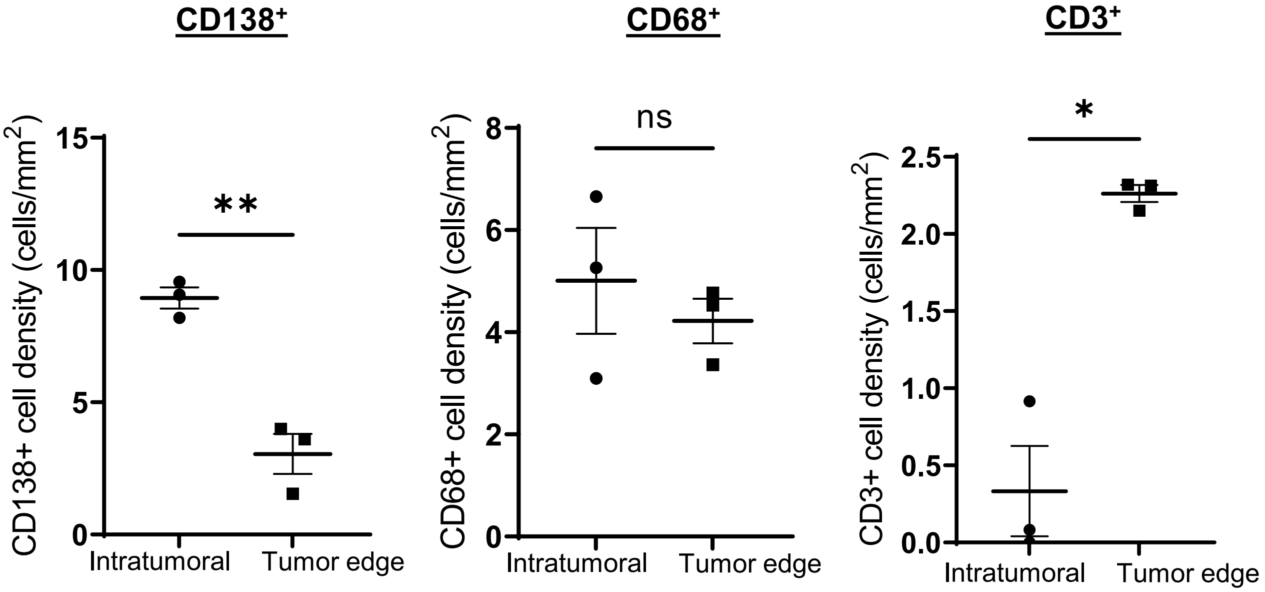


**Supplemental Figure 8. Differences in CD3+ T and CD68+ myeloid cells in areas of tumor clusters with immune exclusion.**

Graphs showing density of $CD138^+$, $CD68^+$ and $CD3^+$ T cells in 3 representative regions within tumor (intratumoral) and outside the tumor (tumor edge) as shown in Fig1B. Figure shows mean ± SEM. Unpaired t test with Welch's correction. *p<0.05, **p<0.01, ns; not significant

Supplemental Figure 9

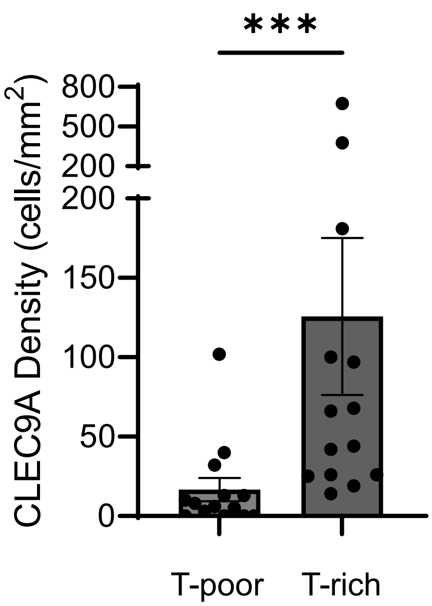


**Supplemental Figure 9. Enrichment of CLEC9A+ DCs in T-cell rich areas.** Analysis of 57 regions of interest (ROIs) from 7 FFPE patient samples was performed. Data was sorted by density (cells/mm$^2$) of CD3$^+$ cells. Upper (T-rich) and lower (T-poor) CD3$^+$ cell density quartiles are represented in this chart with the density of CLEC9A$^+$ cells quantified. Figures show mean ± SEM. Each dot represents a distinct area of a patient sample. Two-tailed unpaired t test with Mann-Whitney test, ***p=0.0002
.

Supplemental Figure 10

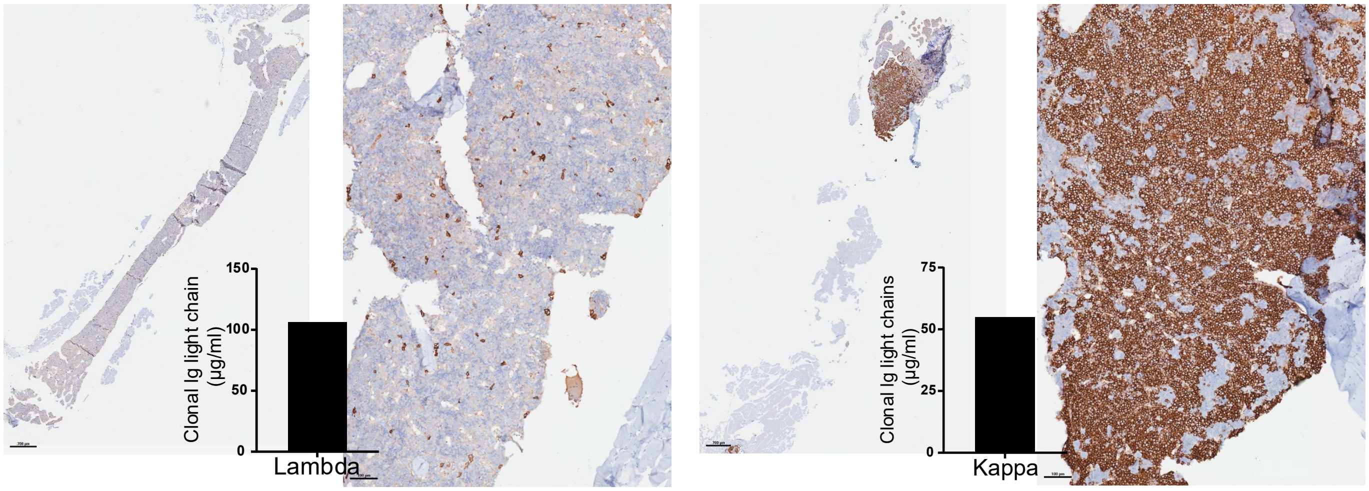


**Supplemental Figure 10**. **Differences in the pattern of tumor growth for MGUS or MM in MISTRG6 mice**.

MISTRG6 mice were injected intrafemorally with tumor cells from patients with MGUS or MM. Mice were euthanized at comparable levels of cloned Ig in serum (inset) and whole bone mounts were analyzed for the pattern of tumor growth.

Supplemental Figure 11

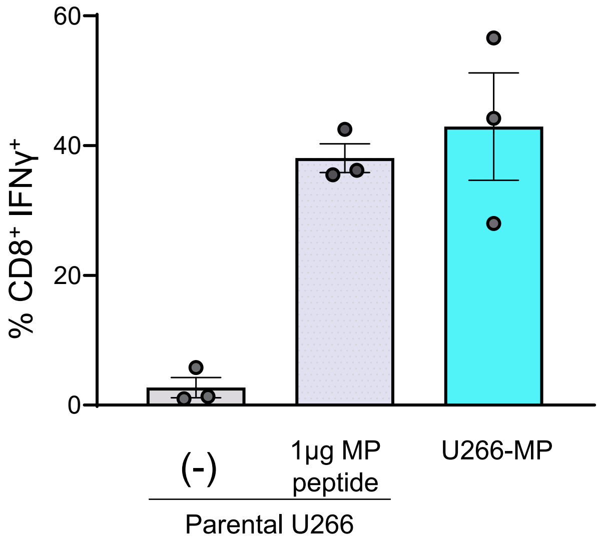


**Supplemental Figure 11. Recognition of U266-MP cells in suspension cultures by MP-specific CD8⁺ T cells, as determined by IFNγ intracellular cytokine staining (ICS).**

MP-specific T cells were cocultured with U266 cells engineered to express MP (U266-MP). Parental U266 MM cells with or without exogenously pulsed MP peptide served as a control. The expression of IFNγ by A2-MP-tetramer⁺ CD8⁺ T cells was monitored by flow cytometry. Figure shows mean ± SEM. Unpaired t test with Welch's correction. *$p<0.05$

Supplemental Figure 12

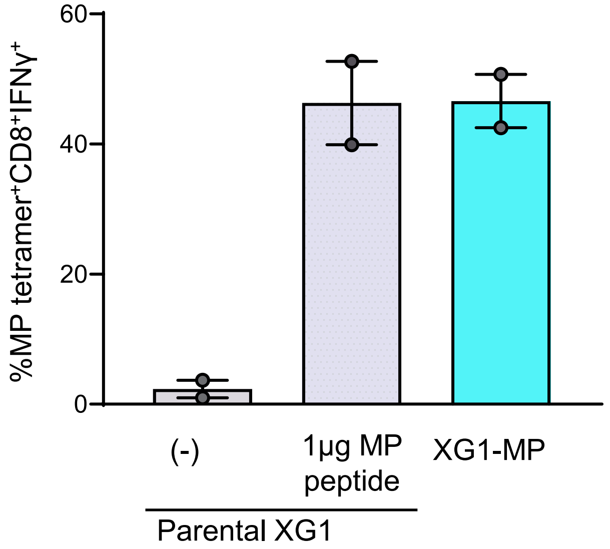


**Supplemental Figure 12. Recognition of MP expressing HLA A2$^+$ XG1 MM cells.**

HLA A2$^+$ XG1 myeloma cell line was engineered to express A2-specific influenza matrix peptide (XG1-MP) and recognition of XG1-MP cells in suspension cultures by MP-specific CD8$^+$ T cells was determined by IFNγ intracellular cytokine staining (ICS). Expanded MP T cells from HLA A2$^+$ healthy donor were co-cultured with XG1-MP cells or parental XG1 cells alone or pulsed with influenza MP as control. The expression of IFNγ by MP-tetramer$^+$ CD8$^+$ T cells was monitored by flow cytometry. Figure shows mean ± SEM. Unpaired t test with Welch's correction.

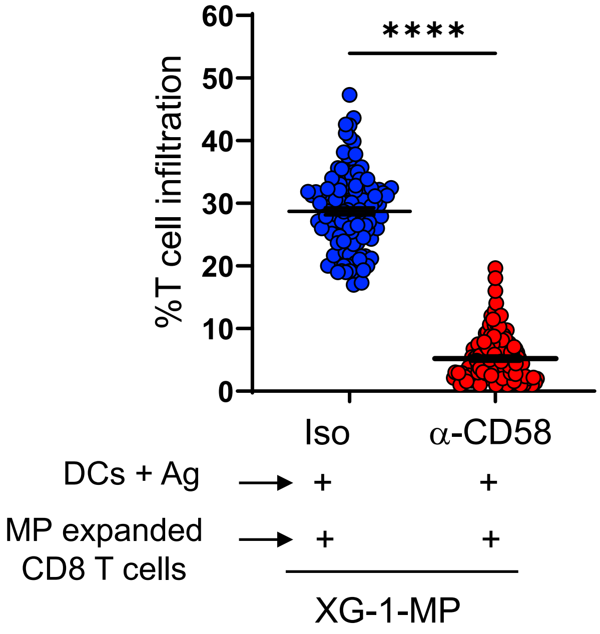


**Supplemental Figure 13. Effect of CD58 blockade on the entry of antigen-specific T cells in XG-1-MP tumor clusters.**

XG-1-MP colonies treated with either anti-CD58 blocking antibody (red) or IgG1 isotype control (blue) were injected with MP-pulsed DCs. MP-specific T cells were added to the colonies and the entry of antigen-specific T cells into clusters was quantified after overnight culture using microscopy. Each dot represents a tumor cluster and data are pooled from a minimum of 3 repeated experiments. Figure shows mean ± SEM. Two-tailed unpaired t test with Welch's correction, ****$p<0.0001$

Supplemental Figure 14

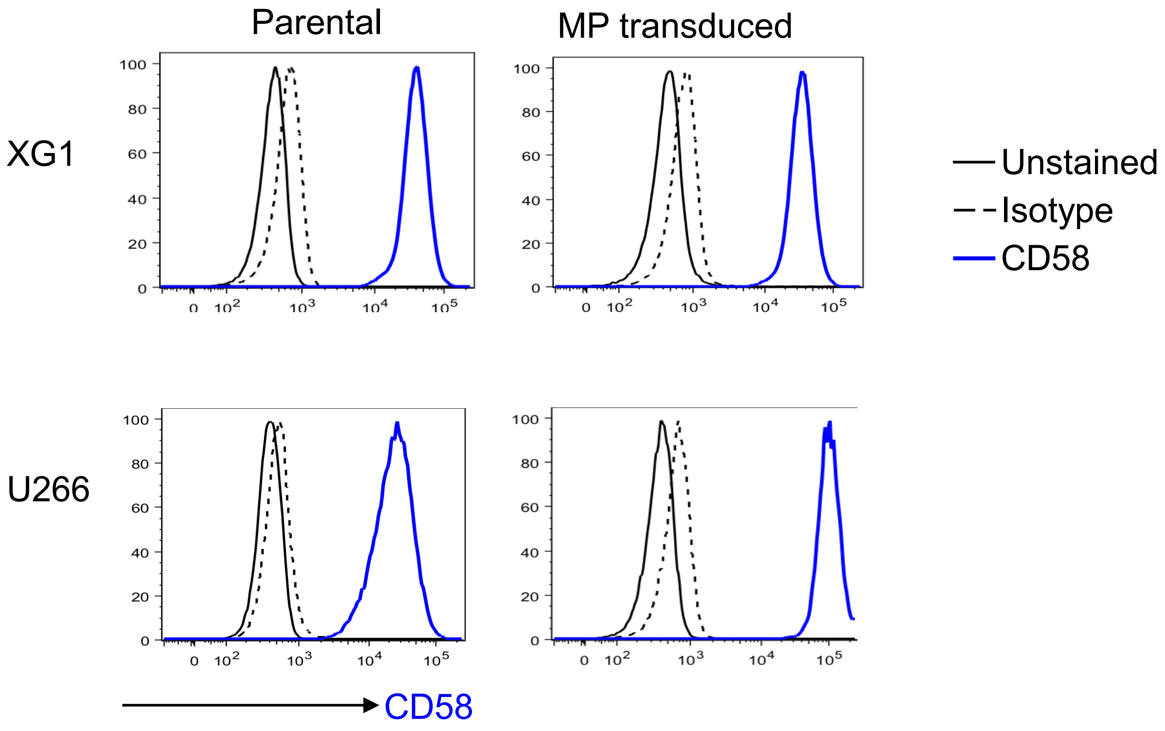


**Supplemental Figure 14. Expression of CD58 by MM cell lines**

Figure shows expression of CD58 on parental and MP transduced U266 and XG-1 myeloma cell lines.

Supplemental Figure 15

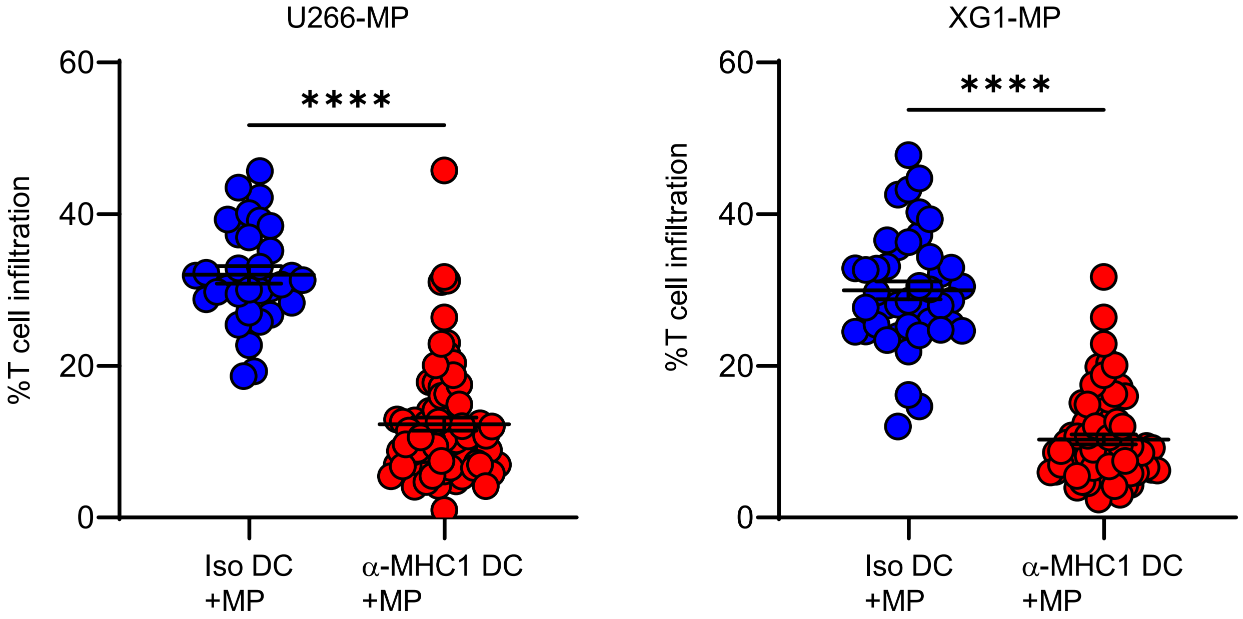


**Supplemental Figure 15. Blockade of major histocompatibility complex 1 (MHC-1) abrogates T cell infiltration of tumor clusters.** MP transduced U266 (U266-MP) and MP transduced XG1 (XG-1-MP) tumor clusters were generated in MethoCult media. Mature DCs were treated with 50 µg/mL α-MHC-1 or isotype control for 1 hour and then loaded with 1 µg/mL Flu-MP (αMHC1 DC+MP and IsoDC+MP respectively). The DCs were added to the colonies and incubated for 4hrs. Fluorescently labeled MP-expanded autologous CD8$^{+}$ T cells were added to the colonies and imaging was performed using a confocal microscope after 24 hours. Each dot represents a distinct tumor cluster and data are pooled from a minimum of 3 repeated experiments. Mann-Whitney t-test was performed to generate the p values. ****p<0.0001

Supplemental Figure 16

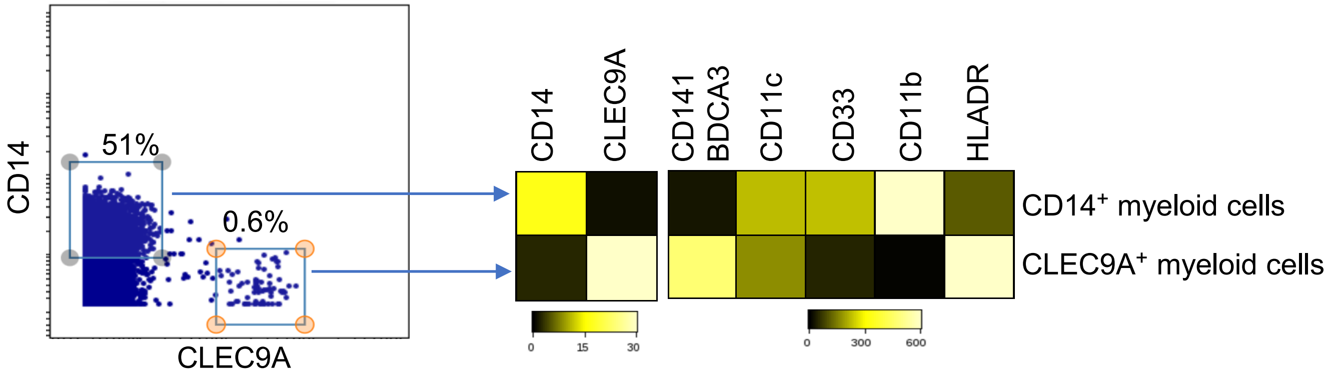


**Supplemental Figure 16. Immunophenotype of CLEC9A$^{+}$ cells**

Bone marrow mononuclear cells obtained from myeloma patient were examined using single cell mass cytometry to characterize myeloid cells. Dot plot gated on CD3$^{-}$/CD19$^{-}$/CD56$^{-}$/CD45$^{high}$ cells shows that CLEC9A$^{+}$cells are a distinct CD14$^{-}$ myeloid population. Heat map on the right shows further characterization showing these CLEC9A$^{+}$ myeloid cells are CD14$^{-}$, CD33$^{-}$, and CD11B negative and instead express CD141 (BDCA3) and are HLA-DR$^{high}$ as previously described for human CLEC9A$^{+}$ DCs.

Supplemental Figure 17

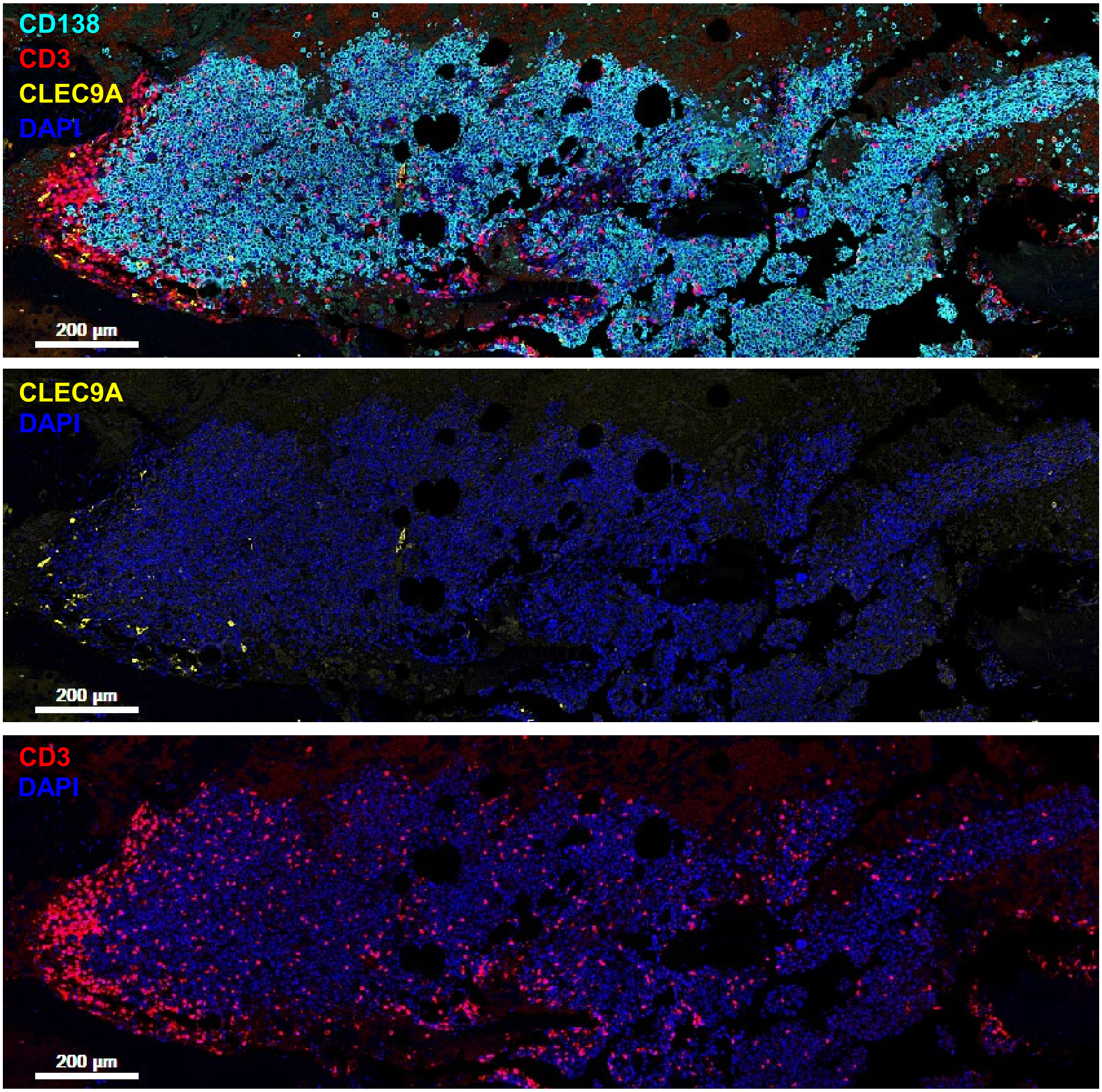


**Supplemental Figure 17. Distribution of CLEC9A⁺ DCs**

Low power images (4x) of an example MM case illustrating CLEC9A⁺ dendritic cells at the edge of a dense tumor cluster and the presence of CD3⁺ T cells in close proximity to these CLEC9A⁺ DCs.

Supplemental Figure 18

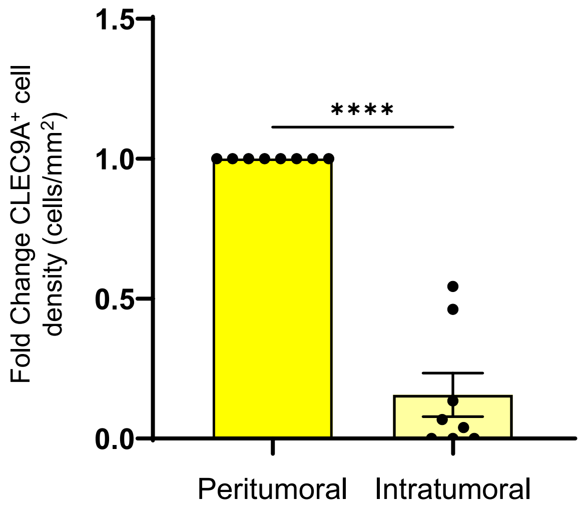


**Supplemental Figure 18**. **Distribution of CLEC9A⁺ DCs relative to tumor clusters**.

Plot showing fold change of the density (cells/$mm^2$) of CLEC9A$^+$ cells in intratumoral areas relative to peritumoral areas . Figure shows mean ± SEM. Each dot represents a gradient zone in a region of interest (8 regions from 6 patient samples). Unpaired t test with Welch’s correction, ****p<0.0001

Supplemental Figure 19

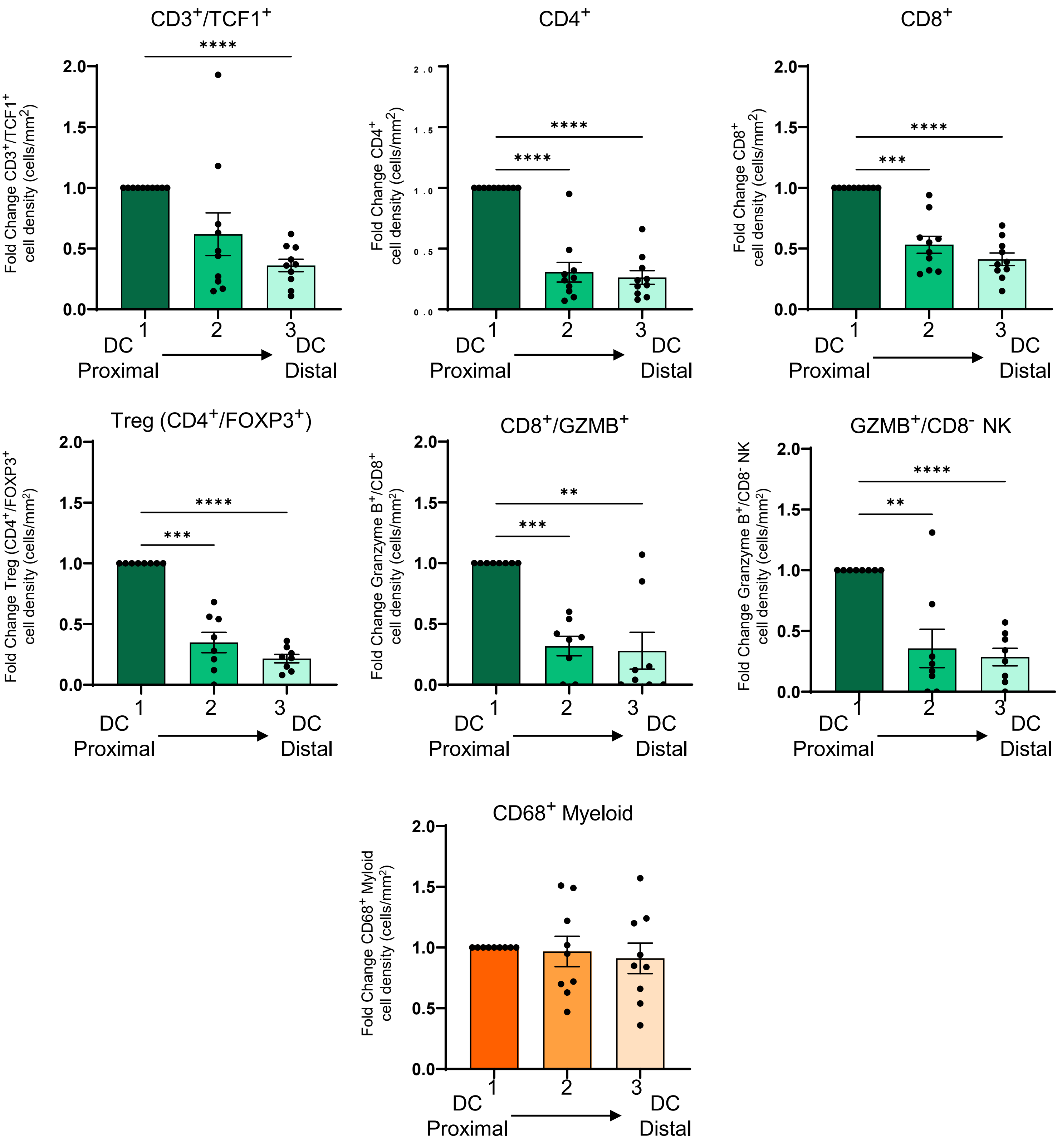


**Supplemental Figure 19. Distribution of immune cells in relation to CLEC9A$^{+}$ DCs.**

Please refer to main figure 4. Gradients from CLEC9A$^{+}$ DCs included several T cell subsets (CD4$^{+}$, CD8$^{+}$, CD3$^{+}$/TCF1$^{+}$, CD4$^{+}$/FOXP3$^{+}$ Treg and CD8$^{+}$/GZMB$^{+}$ T cells), as well as GZMB$^{+}$/CD8- NK cells, but not CD68$^{+}$ myeloid cells. Graphs represent fold change of density of immune cells relative to CLEC9A$^{+}$ DC proximal region. Figures show mean ± SEM. Each dot represents a gradient zone in a region of interest (8 - 10 regions from 6 patient samples).RM one-way ANOVA, with the Geisser-Greenhouse correction and Dunnett's multiple comparisons test, with individual variances computed for each comparison. *p<0.05, **p<0.01, ***p<0.001, ****p<0.0001

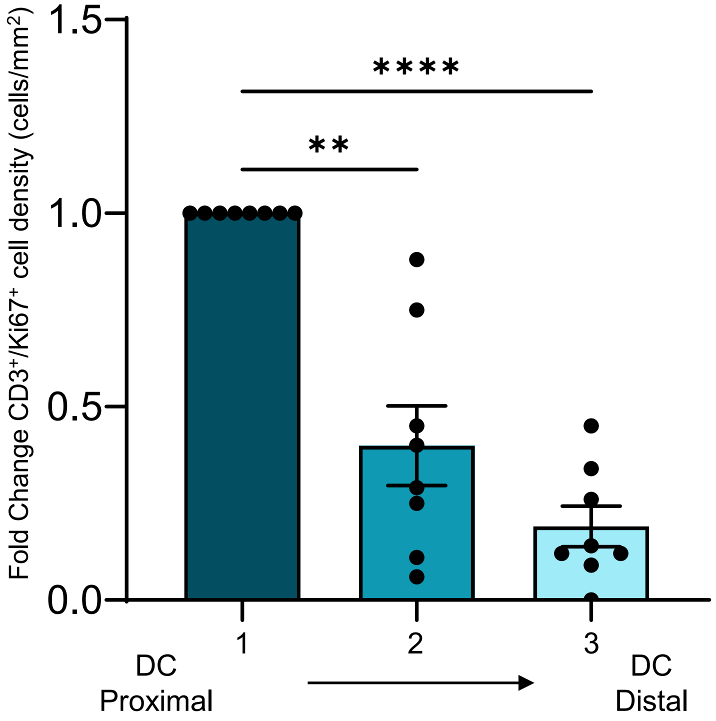


**Supplemental Figure 20. Expression of Ki67 in T cells relative to DC proximity.**

A small subset of MM cases (*n* = 5) was stained using a modified BM1 staining panel (See Supplemental Figure 2) with an antibody for Ki67 replacing the antibody for CD68. Resulting images were analyzed using HALO (Indica labs). As in Figure 4, T cell density was measured in tumor clusters in regions proximal or distal to CLEC9A$^+$ DCs. Graph shows fold change of density of Ki67 on T cells in the context of proximity to CLEC9A$^+$ DCs. Figure shows mean ± SEM. Each dot represents a gradient zone in a region of interest (8 regions from 5 patient samples). RM one-way ANOVA, with the Geisser-Greenhouse correction and Dunnett's multiple comparisons test, with individual variances computed for each comparison. **$p<0.01$, ****$p<0.0001$

Supplemental Figure 21

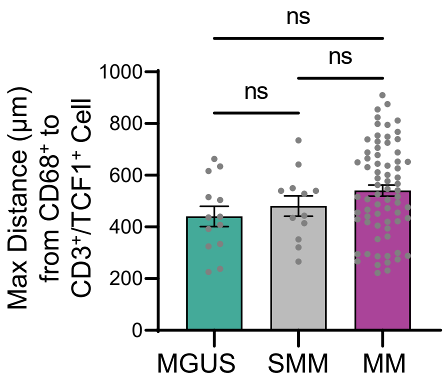


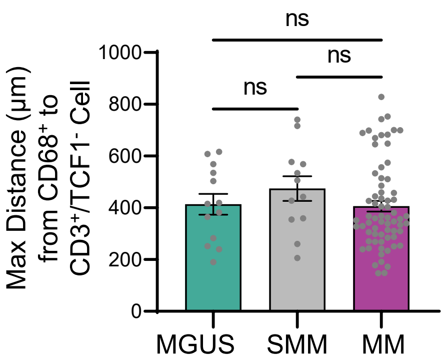


**Supplemental Figure 21**. Maximum distance between CD68$^{+}$ myeloid cells and TCF1$^{+}$ or TCF1$^{-}$ T cell subsets in MGUS (*n* = 13), SMM (*n* = 12) and MM (*n* = 70) cohorts. Figures show mean ± SEM. Brown-Forsythe and Welch ANOVA tests with Dunnett's T3 multiple comparisons test.

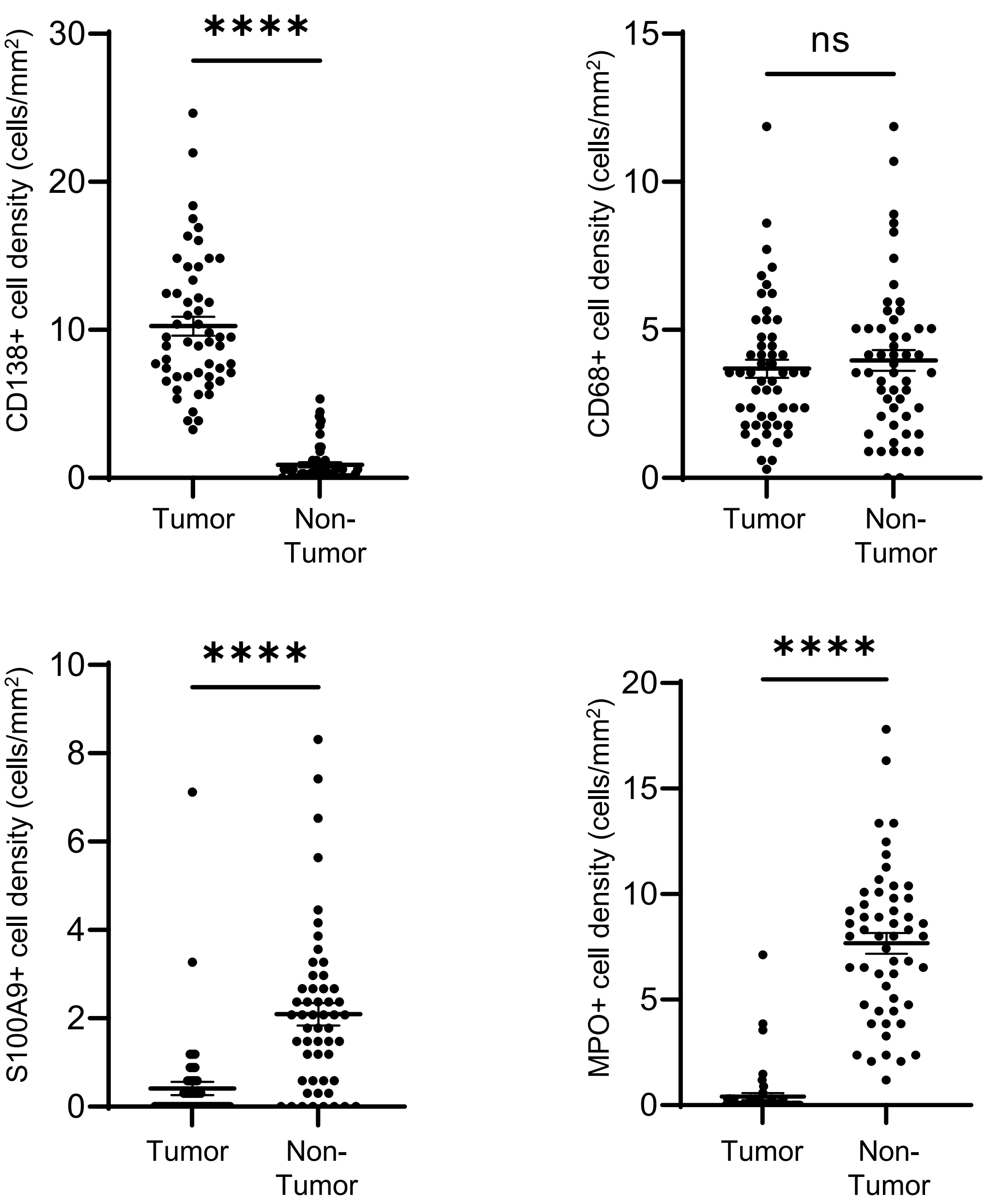


**Supplemental Figure 22. Distribution of A100A9+ and MPO+ cells relative to tumor**

Using CD138 as a guide, identically sized areas of dense CD138 (tumor) and an equal number of areas with sparse CD138 (non-tumor) were selected for each patient sample (*n* = 5). A total of 106 areas, 53 each for tumor and non-tumor were analyzed using HALO (Indica Labs). Density (cells/mm$^2$) was calculated. Figures show mean ± SEM. Each dot represents a distinct area of a patient sample. Two-tailed unpaired t test with Welch's correction, ****p<0.0001

Supplemental Figure 23

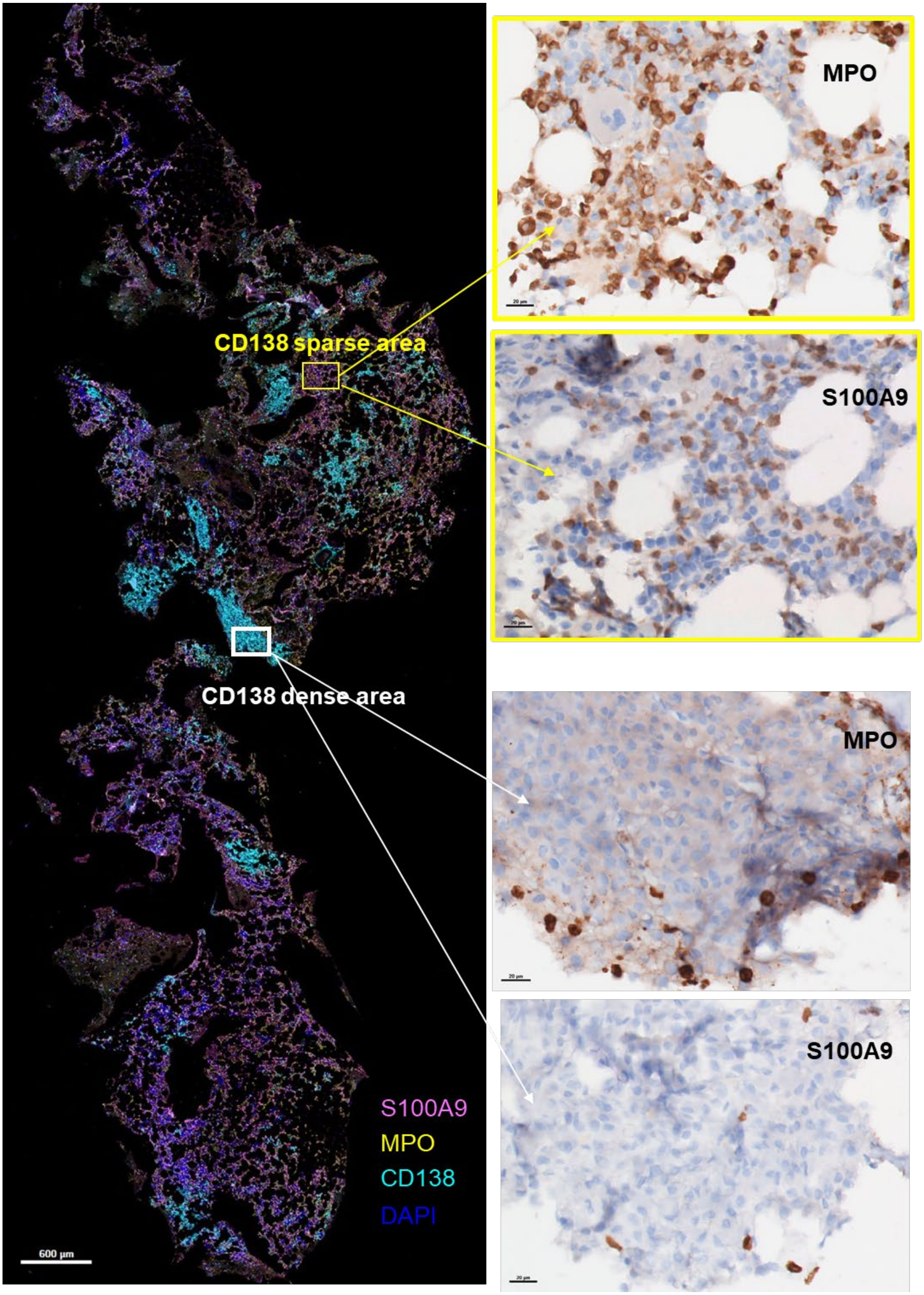


**Supplemental Figure 23. Pattern of S100A9⁺ and MPO⁺ cells.**

Left panel shows multiplex staining. Right panels show confirmation of staining with single color chromogenic assay. Both cell types are mostly in tumor-sparse regions indicating that lack of staining in tumor-rich areas in not due to antibody interference in mIF.

Supplemental Figure 24

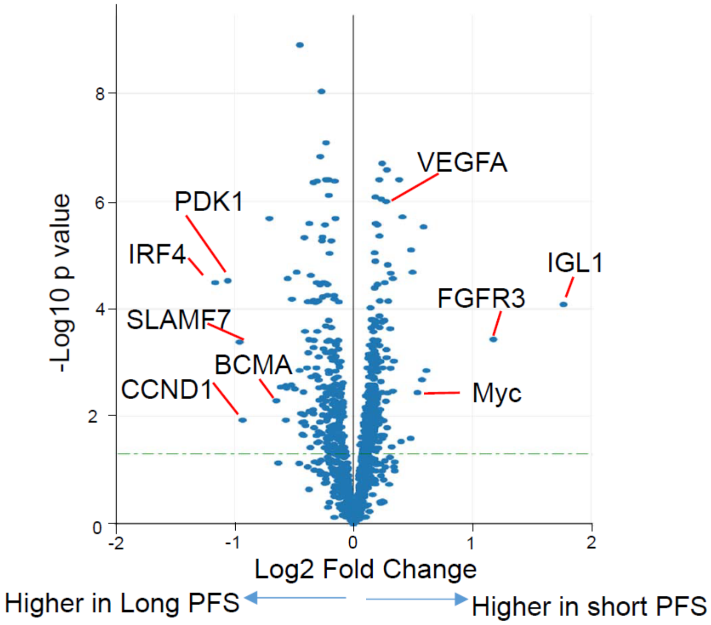


**Supplemental Figure 24.** Volcano plots in ROIs from patients with short (<2 yr; 47 ROIs) versus long (>5 yr; 50 ROIs) PFS. Dotted green line shows corrected p value threshold.
