# Supplementary material for: Regulation of antigen-specific T cell infiltration and spatial architecture in multiple myeloma and premalignancy
Source: J Clin Invest. 2023 Aug 1;133(15):e167629. doi: 10.1172/JCI167629 (PMC10378152; doi:10.1172/JCI167629)
Supplement: Supplemental table 1 [file jci-133-167629-s010.pdf]

Supplemental Table 1. Clinical Characteristics

Patient Characteristics			
		N = 70	%
Median Age , range (years)		58.8 (31.2-79)	
Gender	Male	40	57.1
	Female	30	42.9
Race	White	43	61.4
	Black	24	34.3
	Asian	3	4.3
Risk	Standard	50	71.4
	High	20	28.6
	t(4;14)	6	8.6
	t(14;16)	8	11.4
	t(11;14)	13	18.6
	del 17p	8	11.4
	amp 1q	24	34.3
Isotype	IgG	43	61.4
	IgA	10	14.3
	IgM	1	1.4
	Light chain only	15	21.4
	Non-secretory	1	1.4
ISS	stage I	25	35.7
	stage II	19	27.1
	stage III	12	17.1
	Missing	14	20
Induction Therapy	RVD	69	98.6
	VDD	1	1.4
Stem cell Transplant	Yes	60	85.7
	No	10	14.3
Maintenance Therapy	Yes	64	91.4
	No	6	8.6

Abbreviations: RVD: Revlimid, velcade and dexamethasone; VDD: Velcade, daratumumab and dexamethasone; ISS: International staging system
